# Cell Polarization and Epigenetic Status Shape the Heterogeneous Response to Type III Interferons in Intestinal Epithelial Cells

**DOI:** 10.3389/fimmu.2017.00671

**Published:** 2017-06-12

**Authors:** Sudeep Bhushal, Markus Wolfsmüller, Tharini A. Selvakumar, Lucas Kemper, Dagmar Wirth, Mathias W. Hornef, Hansjörg Hauser, Mario Köster

**Affiliations:** ^1^Research Group Model Systems for Infection and Immunity, Helmholtz Centre for Infection Research (HZI), Braunschweig, Germany; ^2^Institute for Medical Microbiology, RWTH Aachen University Hospital, Aachen, Germany

**Keywords:** epithelial cell line, interferon-lambda, heterogeneous gene expression, cell polarization, small intestinal organoids

## Abstract

Type I and type III interferons (IFNs) are crucial components of the first-line antiviral host response. While specific receptors for both IFN types exist, intracellular signaling shares the same Jak-STAT pathway. Due to its receptor expression, IFN-λ responsiveness is restricted mainly to epithelial cells. Here, we display IFN-stimulated gene induction at the single cell level to comparatively analyze the activities of both IFN types in intestinal epithelial cells and mini-gut organoids. Initially, we noticed that the response to both types of IFNs at low concentrations is based on a single cell decision-making determining the total cell intrinsic antiviral activity. We identified histone deacetylase (HDAC) activity as a crucial restriction factor controlling the cell frequency of IFN-stimulated gene (ISG) induction upon IFN-λ but not IFN-β stimulation. Consistently, HDAC blockade confers antiviral activity to an elsewise non-responding subpopulation. Second, in contrast to the type I IFN system, polarization of intestinal epithelial cells strongly enhances their ability to respond to IFN-λ signaling and raises the kinetics of gene induction. Finally, we show that ISG induction in mini-gut organoids by low amounts of IFN is characterized by a scattered heterogeneous responsiveness of the epithelial cells and HDAC activity fine-tunes exclusively IFN-λ activity. This study provides a comprehensive description of the differential response to type I and type III IFNs and demonstrates that cell polarization in gut epithelial cells specifically increases IFN-λ activity.

## Introduction

The innate defense against viral infection in mammals is based on the coordinated action of type I and type III interferons (IFNs), which are produced by virus-infected and bystander cells (1–3). IFNs induce antiviral mechanisms within virus-infected and uninfected cells and contribute to the adaptive immune responses against viral pathogens (4, 5). Both IFN types reprogram gene expression through the same signal transduction pathway involving the formation of the ternary ISGF3 complex, composed of STAT1, STAT2, and IRF9 (6). Following nuclear translocation, the ISGF3 complex binds to the promoters of IFN-stimulated genes (ISGs) and regulates gene transcription. Thus, type I and type III IFNs induce the expression of a highly overlapping set of genes and share biological activities in the affected cells (2, 7–10). A major difference between the type I and type III IFN-mediated antiviral systems is their engagement of different receptor chains. Whereas all type I IFNs utilize a heterodimeric receptor complex composed of IFN-αR1 and IFN-αR2 subunits, type III IFNs engage the IFN-λR1 (also known as IL28R) and IL10R2 receptor chains for signaling (2, 3). This allows a tissue- and cell-type-specific response. While the type I IFN receptor is found ubiquitously, expression of the IFN-λ receptor is mainly restricted to the epithelium of mucosal surfaces and also to a few other cell types such as hepatocytes in humans (11). Because most pathogens enter the host through mucosal surfaces, the IFN-λ-based antiviral response is the determining factor to establish the first line of defense against invading pathogens (12–14).

Apart from graded responses toward different concentrations of external stimuli, cells can adopt a metastable state with respect to the initiation of signaling events and show bimodal forms of responses. This generates a heterogeneous response within a cell population. This heterogeneity is a hallmark of embryonic cells and was shown to correlate with cell-specific patterns of transcription factor expression and chromatin modifications. While this heterogeneity has been extensively studied in stem cells during embryonic development (15), bimodal responses toward external signals were also found in differentiated cells as exemplified by immune responses to PAMPS or cytokines (16–18). Rand et al. demonstrated that type I IFNs, in particular at low concentrations, lead to the induction of ISGs and subsequent establishment of an antiviral state only in a fraction of cells of a clonal population, whereas others do not respond at all (19).

Although the type I and type III receptor complexes induce the same Jak-STAT signaling, they are structurally distinct and might thus exhibit differences in their signal propagation (20). Since the strength and kinetics of gene induction from type I and type III IFNs differ, we aimed at comparing signal transduction and gene activation in a controlled setting. We employed a recently established murine intestinal epithelial cell line (IEC) (21) and gut stem cell organoids generated from a transgenic fluorescent IFN response reporter mouse. Both culture systems are responsive to both types of IFNs and show properties such as cell polarization and differentiation that reflect critical functional aspects of the gut epithelium *in vivo* (21, 22). The use of the fluorescent reporter allowed us to monitor ISG induction at the cellular level and record the heterogeneity of responses to both IFNs in real time. Indeed, both types of IFNs installed a bimodal distribution of ISG expression within a clonal population. The extent of intrinsic heterogeneity was strongly manifested at low IFN concentrations and depended for IFN-λ on the cellular polarization status. The digital response was based on stochastic decisions downstream of STAT1 nuclear translocation, presumably at the transcriptional level within individual cells. Further experiments highlighted the importance of histone deacetylase (HDAC)-mediated epigenetic modifications during IFN-λ but not during type I IFN induction. Our results demonstrate significant differences in the response toward type I and type III IFNs and identify cell polarization and epigenetic modifications as underlying responsible mechanisms.

## Materials and Methods

### Generation of the Bacterial Artificial Chromosome (BAC) Mx2tRFP

The BAC clone RP24-71I6 containing the murine Mx2 locus was obtained from BACPAC resource center. Homologs recombination was performed using the bacteriophage λ recombination system (23). Thereby, the open reading frame of the murine Mx2 gene was replaced by a linear fragment containing the amplified reporter TurboRFP (Evrogen) followed by an SV40 polyadenylation signal and an FRT (FLP recognition target) flanked cassette harboring a prokaryotic promoter, the PGK-promoter, a gene encoding for kanamycin/neomycin phosphotransferase and the bovine growth hormone polyadenylation signal. Primers used: Mx2Phom+Fluc2: 5′-TTA TAA TAT TCA TTT CCC ACA GAG TAC CCA ACT GAG AGA AGA AAT AAA AGA TGG AAG ATG CCA AAA ACA TTA AGA-3′ and Mx2Exon14hom+*Bam*HI: 5′-AAA GAA AAG TGG TTT ATT AAG GAA TGC AAC AGG CAG CTC CCA TTT GTA CAC TCA AGG GCA TCG GTC GAC GGA TCC-3′. Modified BAC DNA was isolated using NucleoBond BAC100 (Macherey-Nagel).

### Cell Lines, Virus Infection, and Reagents

The intestinal epithelial cell line IEC-Mx2Luc-10 was generated from a transgenic mouse containing the firefly luciferase gene under the transcriptional control of the Mx2 promoter region as described earlier (21). The cell line IEC Mx2tRFP was established by transfecting the BAC Mx2tRFP into IEC-Mx2Luc-10. After selection, clones were picked and tested for similarity in morphology, barrier formation, and reactivity to type I and type III IFNs compared to those of the parental cell line. A representative cell clone showing stable expression of the reporter and efficient barrier formation indicated by an increase in the trans-epithelial electrical resistance (TEER) was selected. IECs were stimulated with IFN-β or IFN-λ3 (PBL Assay Science) and treated with the HDAC inhibitors valproic acid (VPA) (750 µM, Sigma-Aldrich), TSA (2 µM, Sigma-Aldrich), and MS275 (0.51 or 1.7 µM, Selleckchem) and the Bromodomain inhibitor I-BET151 (250, 500, or 800 nM, Cellagentech) as described in the figure legends. IECs were pre-stimulated with IFN-β or IFN-λ3 for 24 h. Infection with vesicular stomatitis virus (VSV) containing an EGFP reporter (24) was performed after washing with serum-free medium. After 1 h of infection, residual virus was removed by washing three times with serum-containing medium.

### Barrier Formation and Polarization

Intestinal epithelial cell lines were grown until fully polarized in transwell cultures as already described earlier (21). 3 × 10^5^ cells were grown on 0.4 µM pore sized transwell inserts (Costar). The culture medium was renewed every third day. The TEER was measured by a chopstick electrode with Volt/Ohm meter (World Precision Instruments). TEER values are reported as Ω*cm^2^, i.e., the resistance in Ohm multiplied by the surface area of the transwell insert. The resistance value for the transwell insert without cells was subtracted as the basal resistance.

### Organoid Derivation and Cultivation

Organoids were cultured from crypt-enriched jejunal and ileal fractions from 6- to 12-week-old Mx2tRFP mice as previously described (22). Briefly, a 10 cm midsection of the small intestine was excised and flushed with ice cold PBS. After removal of mucus and villi, the intestine was cut into 1–2 cm pieces and washed extensively with cold PBS. The epithelium was dissociated for 30 min at 4°C in a solution of 2 mM EDTA in PBS. Afterward, the crypts were suspended in 10% FCS in PBS and passed through a 70-mm cell strainer (BD Biosciences), centrifuged at 200 *g* (5 min, 4°C), and resuspended in 10 ml Ad-DF medium [advanced DMEM/F12 supplemented with 1% Glutamax (Invitrogen), 10 mM HEPES, and 100 U/ml of Penicillin/Streptomycin]. After centrifugation, the crypts were resuspended in Matrigel (BD Biosciences) at a desired crypt density. 20 µl Matrigel was seeded per well on a pre-warmed 48-well flat-bottom plate and incubated for 30 min at 37°C and 5% CO_2_ atmosphere. Then, 300 µl of Intesticult organoid growth medium (Stemcell Technologies) was added. The passaging was performed every 1–2 weeks with a split ratio of 1:3 by harvesting the organoids, mechanic disruption into single crypt domains, and seeding with fresh Matrigel.

### Antibodies and Western Blotting

Primary antibodies for Western blot analysis were purchased from Cell Signaling Technology (STAT1 Antibody #9172; Phospho-STAT1 (Tyr701) (58D6) Rabbit mAb #9167) and from Santa Cruz Biotechnology (β-Actin (ACTBD11B7) sc-81178). For generation of whole cell extracts, cells were lysed in RIPA buffer (10 mM Tris–HCl, pH 7.5, 150 mM Sodium chloride, 1% Triton X-100, 0.1% Sodium dodecyl sulfate, 1% Sodium deoxycholat, 1 mM Dithiothreitol, 1 mM Sodium orthovanadate, 1 mM Sodium fluoride, 1× HALT™ Protease Inhibitor Cocktail). Whole cell extracts were diluted in 4× NuPAGE^®^ LDS Sample Buffer (Invitrogen), and proteins were separated by denaturing SDS-PAGE in a 10% separation gel (10% Acrylamide/Bis (37.5:1), 0.375 M Tris pH 8.8, 0.1% Sodium dodecyl sulfate, 0.001% TEMED, 0.1% Ammonium persulfate). Proteins were transferred to an activated PVDF membrane, and the membranes were washed three times in TBST, blocked with TBST containing 5% milk powder, and probed by incubation with primary antibodies, followed by incubation with a horse-radish peroxidase-conjugated antibody (Amersham). Luminescence signal was detected by either ECL Advance^®^ (Amersham) or ECL Prime^®^ (Amersham) according to the manufacturer’s instructions. Luminescence was measured using the ChemiDoc XRS system and quantified with Quantity One (Bio-Rad) or ImageJ.

### Luciferase Assay

Cells were washed once in cold PBS and incubated with adjusted amounts of reporter lysis buffer (RLB, Promega) at −70°C for 20 min. Cell lysates were assayed for luciferase activity using standard reaction buffer (20 mM glycylglycine, 12 mM MgSO_4_, 1 mM ATP) containing luciferin (Promega) and a single tube luminometer (Lumat LB 9507, Berthold Technologies).

### Chromatin Immunoprecipitation (ChIP)

Chromatin immunoprecipitation analysis was performed using the ChIP-IT High-Sensitivity Kit (Active Motif) in accordance with the manufacturer’s protocol. Cells were grown to near-confluency in a 100-mm culture dish. In brief, after crosslinking, the cell pellet was suspended in Lysis buffer (20 mM Tris–HCl pH8.0, 85 mM KCl, 0.5% NP-40, PMSF, Protease Inhibitor cocktail, 1 µM TSA) and incubated for 10 min on ice. Following centrifugation, nuclei were lysed in Nuclei Lysis buffer (50 mM Tris–HCl pH8.0, 140 mM NaCl, 1% Triton X-100, 0.1% SDS, 1% Na-Deoxycholate, PMSF, Protease Inhibitor cocktail, 1 µM TSA). Bioruptor™ sonicator (Diagenode) was used to shear chromatin. Immunoprecipitation was performed overnight at 4°C with H3K9ac antibody (Active Motif, #39137). Real-time quantitative PCR was performed using SYBR Green I Master Mix (Roche Applied Sciences) in a LightCycler480 II (Roche Applied Sciences) with specific primers. Primers were designed to amplify proximal promoters containing ISRE site(s). IFIT1, 5′-GTCTGTATCCGTTTCAGAGC-3′ (forward), 5′-GAACAGGGAAATCCTTACCC-3′ (reverse); IRF7, 5′-GAAGGGCAGTGAAGAGAAGC-3′ (forward), 5′-GTCACAGGTGTTAATCCAGC-3′ (reverse); Rsad2, 5′-TCACTGCCTTTCCTTGGCTT-3′ (forward), 5′-GCCTGCAAGGATGCAGCTAT-3′ (reverse). Input C_T_ values were adjusted for dilution and used to calculate % input values for immunoprecipitated samples.

### qRT-PCR

RNA was isolated from IECs using RNeasy Mini Kit (Qiagen, Germany) according to the manufacturer’s instructions and quantified with an ND-1000 spectrophotometer (NanoDrop Technologies). Total RNA from intestinal organoids was isolated using Trizol LS reagent (Life Technologies) according to the manufacturer’s instructions. 2 µg of total RNA was used for cDNA synthesis using Ready-To-Go You-Prime First-Strand Beads (GE Healthcare). RT-PCR was run at 58°C annealing temperature using SYBR Green I Master Mix (Roche Applied Sciences) in a LightCycler480 II (Roche Applied Sciences). Data were processed using Light Cycler 480 Software 1.5. The mRNA levels were normalized to those of β-Actin gene. Murine PCR primers for β-Actin (forward primer, 5′-TGG AAT CCT GTG GCA TCC ATG AAA C-3′ and reverse primer, 5′-TAA AAC GCA GCT CAG TAA CAG TCC G-3′), IRF-7 (forward primer, 5′-GAA GAC CCT GAT CCT GGT GA-3′ and reverse primer, 5′-CCA GGT CCA TGA GGA AGT GT-3′), Mx2 (forward primer, 5′-TCA CCA GAG TGC AAG TGA GG-3′ and reverse primer, 5′-CAT TCT CCC TCT GCC ACA TT-3′), Rsad2 (forward primer, 5′-GTC CTG TTT GGT GCC TGA AT-3′ and reverse primer, 5′-GCC ACG CTT CAG AAA CAT CT-3′), Usp18 (forward primer, 5′-CAT CCT CCA GGG TTT TCA GA-3′ and reverse primer, 5′-AAG GAC CAG ATC ACG GAC AC-3′), IFI44 (forward primer, 5′-AAC TGA CTG CTC GCA ATA ATG T-3′ and reverse primer, 5′-GTA ACA CAG CAA TGC CTC TTG T-3′), IFIT1 (forward primer, 5′-TGT TGA AGC AGA AGC ACA CA-3′ and reverse primer, 5′-TCT ACG CGA TGT TTC CTA CG-3′), IL28R (forward primer, 5′-CCC TGT TTC CTG ACA CTC CC-3′ and reverse primer, 5′-TCA GAA AAG TCC AGT GCC CG-3′), and IFNAR2 (forward primer, 5′-CTA TCG TAA TGC TGA AAC GG-3′ and reverse primer, 5′-CGT AAT TCC ACA GTC TCT TCT-3′).

### Flow Cytometry and Immunofluorescent Staining

Intestinal epithelial cell lines were seeded in 12-well plates and treated as previously described (21). Flow cytometry analysis was performed on an LSR-II SORP and FACS-Calibur (BD Biosciences). Fluorescence-activated cell sorting was performed on an ARIA-II SORP (BD Biosciences). Data were processed using FlowJo v7.6.5 (Tree Star, Inc.). For immunofluorescent staining, IECs were seeded in an 8-well chamber slide (ibidi) and treated as described. After fixation with 4% formaldehyde, cells were washed with PBST (0.02% Tween in PBS), blocked with 1% BSA in PBS, and stained with Phospho-STAT1 (Tyr701) (Cell Signaling Technology, 58D6, Rabbit mAb) antibody for 1 h at room temperature. After washing, the samples were incubated with FITC-labeled goat anti-Rabbit antibody for 1 h at room temperature. Fluoroshield with DAPI was added for nuclear staining and fluorophore protection. The samples were examined under a Zeiss 510 Laser Scanning confocal microscope.

### Image and Statistical Analysis

Microscopic picture series were analyzed using ImageJ (NIH, Bethesda, MD, USA) and built-in plugins as well as MTrackJ (E. Meijering). All data analyses were performed using GraphPad Prism v5.04 (Graph Pad Software, La Jolla, CA, USA). Results were presented as mean value ± SEM from triplicates or from numbers indicated in the figure legend. Statistical significance was tested using one-way ANOVA, followed by Tukey’s Multiple Comparison test, the non-parametric unpaired Mann–Whitney *U* test, and Student’s *t*-test. The statistical test used for each analysis is mentioned in the respective figure legend. *P*-values less than 0.05 were considered to be statistically significant.

## Results

### Bimodality of Gene Induction in Responses to Type I and Type III IFNs

In order to investigate the differences in type I and III IFN-mediated signaling, we used a recently described IEC that was derived from a transgenic mouse expressing a firefly luciferase reporter under the transcriptional control of the IFN-dependent mouse Myxovirus resistance gene 2 (Mx2) promoter (21). Stimulation of IECs by either IFN-β or IFN-λ3 revealed a significant increase in total luciferase activity over time (Figure S1A in Supplementary Material). However, IFN-β-mediated Mx2Luc gene induction was rapid and peaked between 6 and 9 h after stimulation, followed by a steady decrease to half-maximal activity at 48 h. In contrast, IFN-λ3 induced a delayed but gradual increase in Mx2-driven luciferase activity for up to 48 h. Maximal Mx2 promoter activity following IFN-λ3 stimulation was 5–6 times lower than that induced by IFN-β (Figure S1B in Supplementary Material).

**Figure 1:**
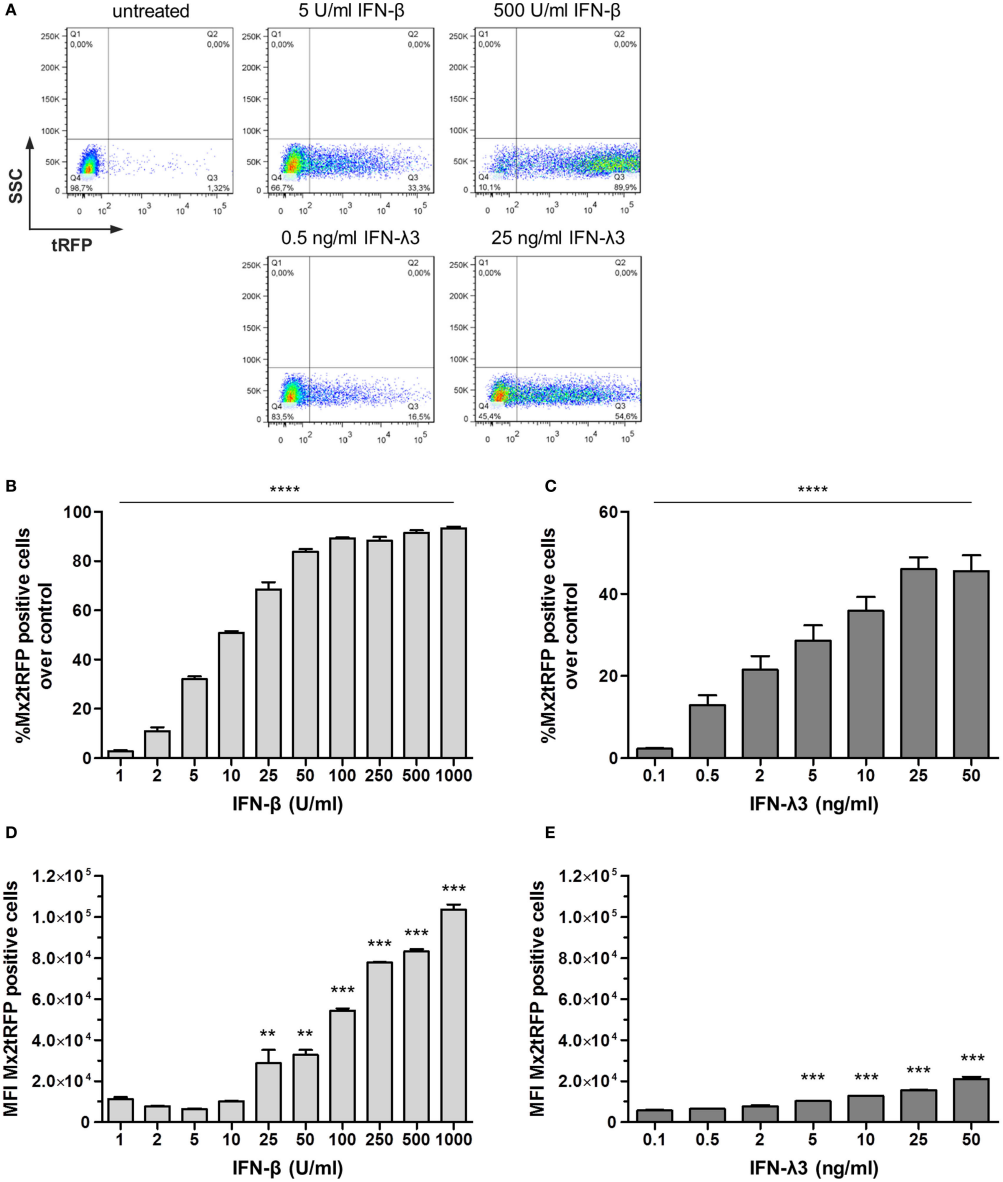
Bimodal nature of gene expression toward type I and type III interferon (IFN) stimulation. Intestinal epithelial cell lines harboring the bacterial artificial chromosome construct Mx2tRFP were stimulated with different concentrations of IFN-β and IFN-λ3 for 20 h. Expression of the tRFP reporter was measured by flow cytometry (*n* = 5–7, mean ± SEM). **(A)** Representative dot plots show Mx2tRFP expression at high and low concentrations of IFN-β and IFN-λ3. **(B,C)** Percentage of Mx2tRFP-positive cells for all used concentrations of IFN-β and IFN-λ3. *P* values were calculated by one-way ANOVA. **(D,E)** Mean fluorescence intensity (MFI) of Mx2tRFP-postive cells. *P* values were calculated by one-way ANOVA, followed by Tukey’s Multiple Comparison Test (*P ≤ 0.05, **P ≤ 0.01, ***P ≤ 0.001). *P* values are given for differences among each stimulated group and control group.

To investigate the timing and dynamic of gene expression in individual cells of a given population, we next generated a clonal IEC line harboring a BAC encoding TurboRFP under the control of the Mx2 promoter region (Mx2tRFP). Mx2tRFP cells were stimulated with increasing concentrations of either type of IFN, and flow cytometric analysis was performed. Stimulation with IFN-β concentrations above 100 U/ml induced Mx2tRFP expression on average in 90% of the cells (Figures 1A,B). In contrast, stimulation with low doses (between 2 and 10 U/ml) of IFN-β left the majority of cells unresponsive and induced Mx2-tRFP expression only in a small fraction of cells. Of note, clonal cell populations were employed for these experiments, indicating that a portion of cells did not properly respond at the time point of IFN stimulation. Mean fluorescence intensity (MFI) determination of the tRFP-positive population indicated a continuous rise in the mean Mx2 promoter activity in individual cells with increasing IFN-β concentrations (Figure 1D). Thus, both the number of cells and the individual cell response increased with increasing IFN-β concentrations. This was also reflected by the continuous increase in Mx2-driven total luciferase activity upon stimulation with increasing doses of IFN-β (Figure S1B in Supplementary Material). Strikingly, stimulation with IFN-λ3 induced the same pattern of digital Mx2tRFP expression characterized by a concentration-dependent gradual increase in the fraction of responding cells. Yet, the tRFP-positive cell fraction even at the highest IFN concentration did not exceed 55% of the total population (Figures 1A,C). Interestingly, only a marginal increase in the MFI of the tRFP-positive population could be observed after IFN-λ3 stimulation (Figure 1E). The reduced ability of high-dose IFN-λ compared to that of IFN-β to induce gene expression in individual cells was confirmed for other ISGs by qRT-PCR (Figure S1C in Supplementary Material). Thus, stimulation of IECs with IFN-λ resulted in Mx2 promoter induction, characterized by a limited number of responding cells and lower levels of gene expression. This is in contrast to type I IFN stimulation, where no such limitations could be observed. As published earlier (8, 25), we observed that the kinetic of ISG induction differs for both IFN types as IFN-λ induces a delayed expression of the Mx2tRFP reporter (Figure S1D in Supplementary Material). Importantly, stimulation with low doses of either IFN-β or IFN-λ resulted in a heterogeneous pattern of Mx2tRFP induction with highly responsive and completely non-responsive subpopulations. This is reminiscent of the IRF-7-mCherry reporter induction by IFN-β in mouse fibroblasts (19).

**Figure 2:**
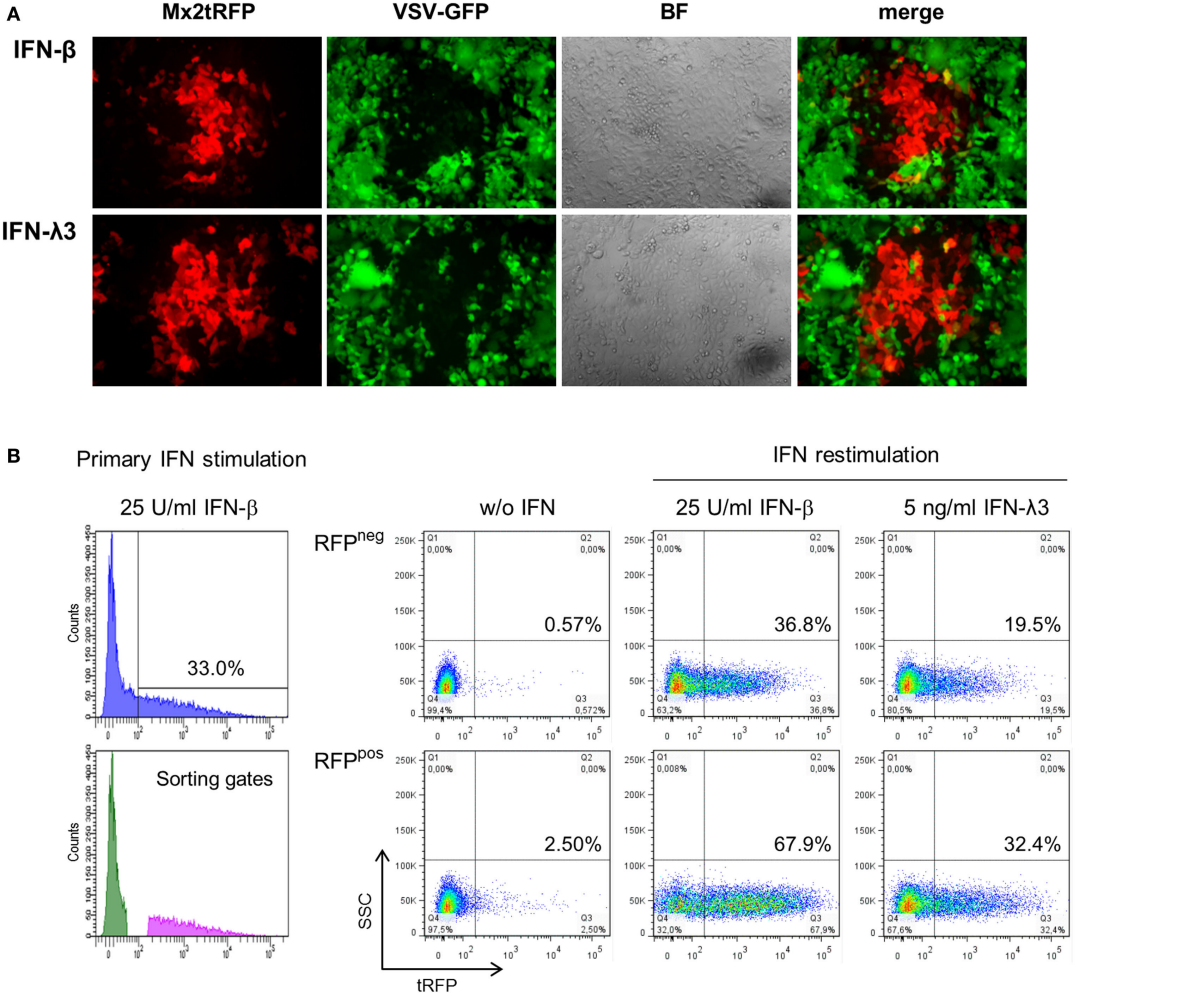

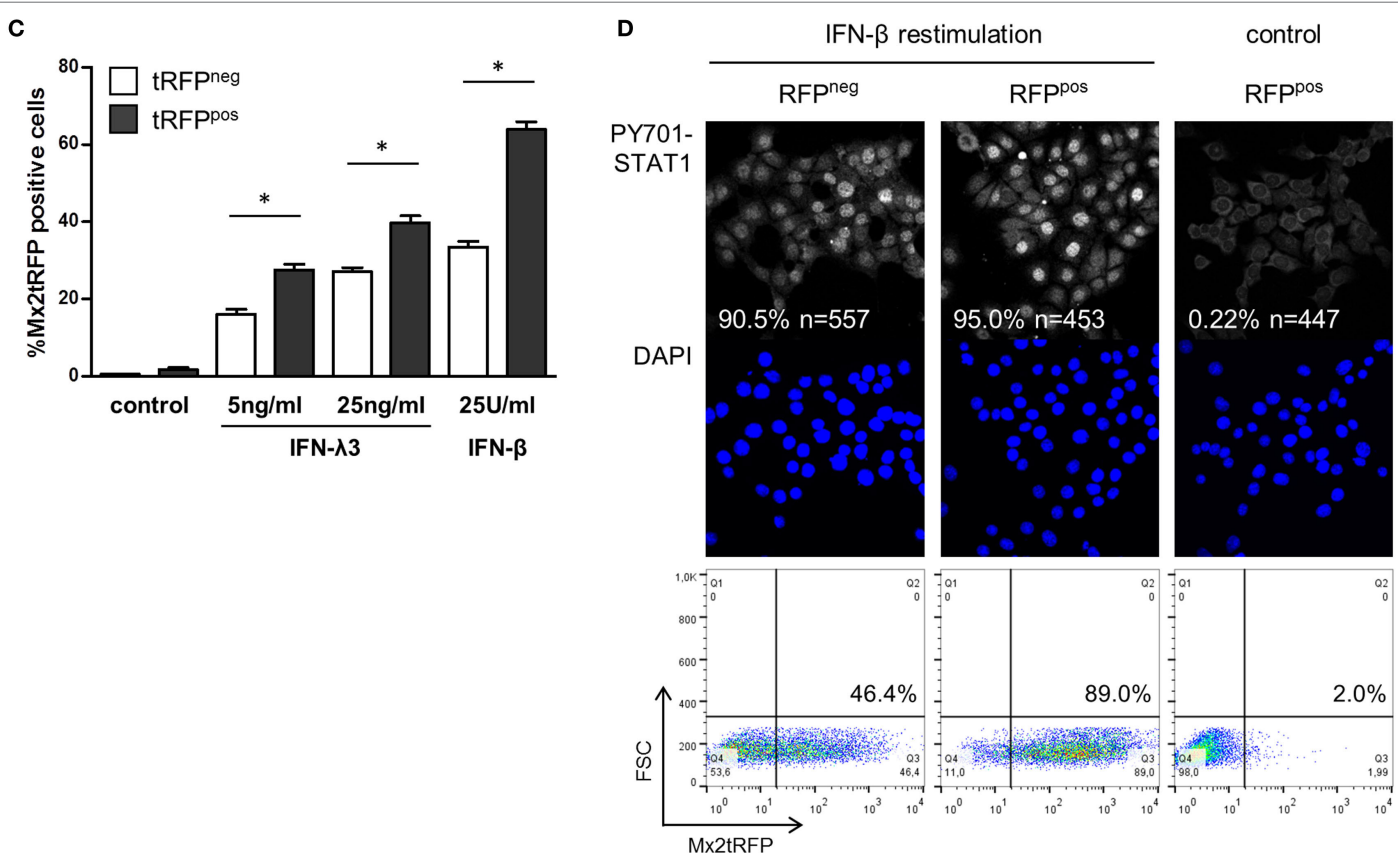
“Memory” effect in IFN-stimulated cells. **(A)** Intestinal epithelial cell lines (IECs) harboring Mx2tRFP were stimulated with either 10 U/ml interferon (IFN)-β or 25 ng/ml IFN-λ3 for 20 h. Cells were infected with vesicular stomatitis virus-GFP (MOI 0.02) for 1 h and imaged by confocal microscopy 20 h post-infection. Representative pictures are shown. Data are representative of three independent experiments. **(B)** IECs were stimulated for 24 h with 25 U/ml IFN-β, and Mx2tRFP-positive and -negative populations were separated by cell sorting. Cells were cultured for 48 h in the absence of IFN, and responder and non-responder populations were restimulated for 20 h with the indicated concentrations of IFN-β and IFN-λ3. Mx2-driven tRFP expression was measured by flow cytometry. Representative FACS dot plots are shown. **(C)** Frequencies of Mx2tRFP expression in responder and non-responder populations upon IFN restimulation were calculated from two independent experiments (*n* = 4, mean ± SEM). *P* values were calculated by Mann–Whitney U test (*P ≤ 0.05). **(D)** Sorted Mx2tRFP-negative and Mx2tRFP-positive cell populations were cultured for 48 h in the absence of IFN. Both populations were stimulated for 1 h with 50 U/ml IFN-β, fixed, and processed by immunofluorescence staining against P-Y701 STAT1. Control column represents Mx2tRFP-positive cells without IFN treatment. Images were obtained by confocal microscopy, and the percentage of cells showing activated STAT1 in the nucleus was calculated (*n* indicates the number of analyzed cells). Representative pictures are shown. DAPI staining indicates localization of cell nuclei. Dot plots show representative Mx2tRFP expression analysis from (B) after restimulation with 50 U/ml IFN-β for 20 h.

Mx2 is one out of many IFN-induced proteins that protect cells against viral infection. To confirm that the observed bimodality of Mx2tRFP expression correlates with the induction of an antiviral state, i.e., reflects the induction of ISGs, IFN-pretreated IECs were subjected to VSV infection. IECs harboring Mx2tRFP were stimulated with either 10 U/ml IFN-β or 25 ng/ml IFN-λ3 for 20 h and subsequently infected with a recombinant VSV constitutively expressing eGFP (VSV-GFP). Fluorescence microscopy revealed that Mx2tRFP-positive cells were protected against infection with VSV, whereas tRFP-negative cells remained susceptible to viral infection (Figure 2A). This inverse correlation between Mx2tRFP expression and VSV-GFP replication was observed for both types of IFNs, IFN-β and IFN-λ3, arguing for comparable thresholds of antiviral activity. Thus, the Mx2 reporter reflects the coordinated induction of at least a group of ISGs sufficient to provide protection against viral replication. These results also indicate that the antiviral state is an unpredictable all-or-nothing decision of individual cells. While higher concentrations of IFN-β lead to a protection of the vast majority of cells within a culture, saturating amounts of IFN-λ3 induced Mx2tRFP expression only in a maximum of 50% of the cells and failed to protect the whole IEC population from viral replication (Figure 2A).

To test whether the bimodal ISG expression pattern during stimulation with low doses of IFN was stable or showed short-term variability, we performed stimulation-sort-restimulation experiments. First, IECs harboring Mx2tRFP were stimulated with a low dose of IFN-β and sorted into non-responding (“non-responder,” tRFP-negative) and responding (“responder,” tRFP-positive) populations (Figure S2A in Supplementary Material). Both populations were cultivated separately for 48 h in the absence of IFN to allow the reporter signal to decrease to baseline levels. Subsequently, cells were restimulated with both types of IFNs, and the Mx2tRFP expression was monitored by flow cytometry. When restimulated, none of the two populations maintained Mx2tRFP expression pattern in the first stimulation. Cells from the non-responder population behaved like naïve cells following primary stimulation with approximately 35% Mx2tRFP-positive cells upon IFN-β exposure (Figures 2B,C). In contrast, the frequency of Mx2tRFP expression and the mean strength of the tRFP signal in the responder cell fraction were highly enhanced upon secondary IFN stimulation. However, approximately 35% of the responder population showed no detectable Mx2tRFP induction upon IFN-β restimulation. Notably, the responsiveness toward restimulation with IFN-λ also increased strongly in the responder population (Figures 2B,C). Restimulation experiments were also conducted with IFN-λ as a primary inducer. Again, upon IFN-λ3 restimulation, Mx2tRFP expression was induced in the non-responding population to frequencies comparable to those of naïve cells, whereas “responder” cells showed a much higher percentage of reporter gene induction (Figure S2B in Supplementary Material). In summary, we conclude that the ability to realize ISG induction in response to low amounts of IFN depends on short-term variables, and we further exclude the existence of a stable fraction of IFN-λ-unresponsive cells within the propagated (clonal) IEC line.

Thus, an intrinsic alteration in the “responder cells” has the capacity to increase the sensitivity toward further IFN stimulation. To address the nature of this intrinsic priming, we determined the frequency of cells with STAT1 nuclear accumulation in each of the responder and non-responder cell populations upon IFN restimulation. The responder and non-responder cell populations were restimulated with 50 U/ml IFN-β for 1 h, fixed, and stained for STAT1-Y701 phosphorylation. Using confocal microscopy, the number of cells staining positive for activated STAT1 in the nucleus was evaluated (Figure 2D). Unexpectedly, the frequency of nuclear phospho-STAT1-positive cells was similar between the tRFP-positive (“responder”) and tRFP-negative (“non-responder”) populations. In both populations, IFN-β stimulation was sufficient to induce nuclear accumulation of Y701-phosphorylated STAT1 in more than 90% of the cells. Thus, IFNAR-dependent STAT1 activation is not significantly influenced by prior IFN stimulation and can thereby be excluded as the underlying mechanism for bimodal cell responses. Instead, it appears likely that the cellular heterogeneity results from alterations at the level of gene transcription, suggesting that epigenetic differences account for the differential reaction toward IFN.

### HDAC Inhibition Enhances the Frequency of Responsiveness upon IFN-λ Stimulation

**Figure 3:**
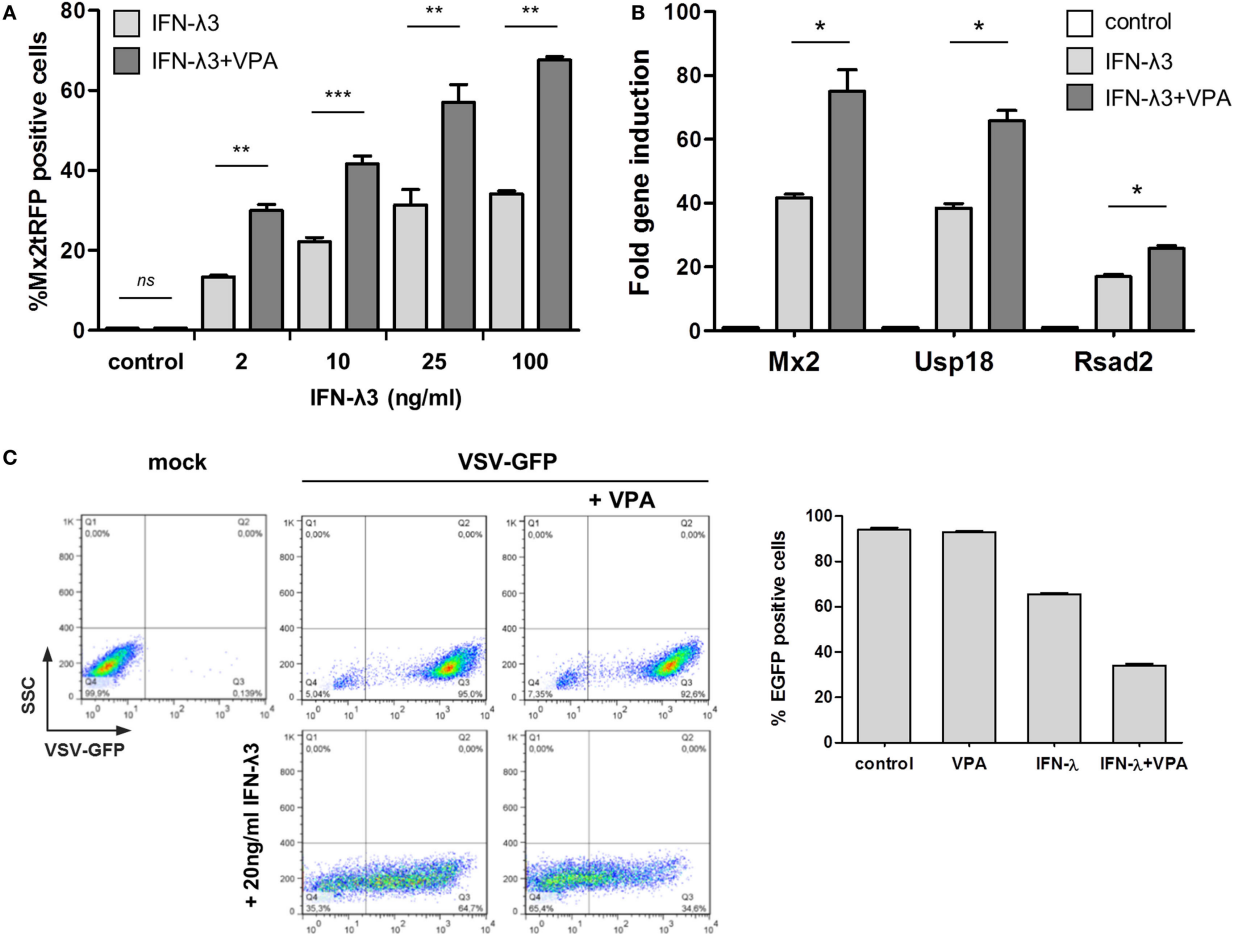

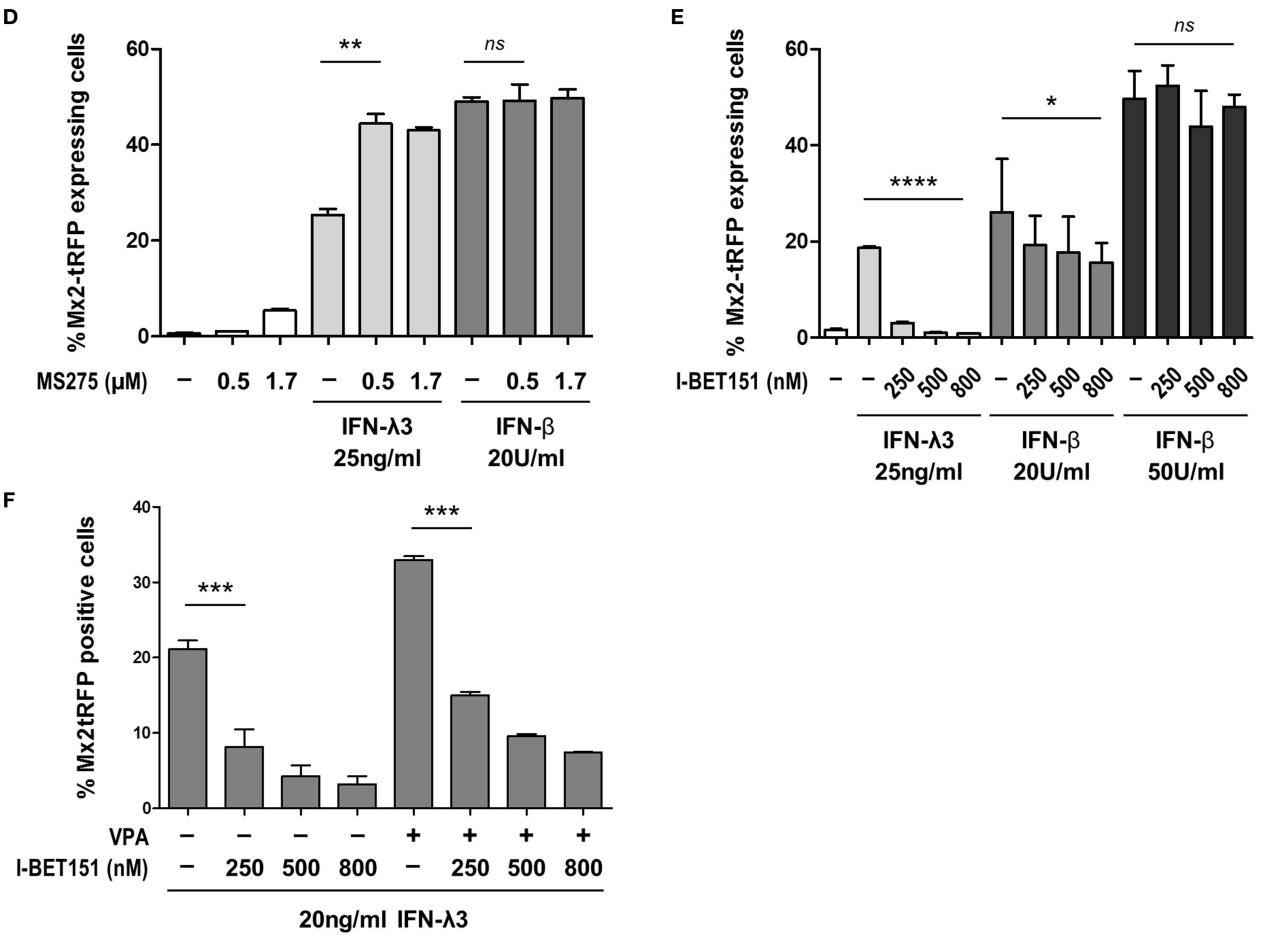
Differential sensitivity of type I and type III interferons (IFNs) to inhibition of histone deacetylase and BRD3/4. **(A)** Intestinal epithelial cell lines (IECs) harboring Mx2tRFP were stimulated with increasing concentrations of IFN-λ3 in the absence or presence of 750 µM valproic acid (VPA). Frequency of Mx2tRFP expression was determined by flow cytometry after 24 h (*n* = 3–6, mean ± SEM). *P* values were calculated by paired *t*-test (*P ≤ 0.05, **P ≤ 0.01, ***P ≤ 0.001). **(B)** IECs were either untreated or treated with 10 ng/ml IFN-λ3 in the absence or presence of 750 µM VPA for 16 h. RNA was isolated, and qRT-PCR was used to determine the expression of Mx2, Usp18, and Rsad2. IFN-stimulated gene expression was normalized to β-Actin (*n* = 3, mean ± SEM). *P ≤ 0.05 by Mann–Whitney *U* test. **(C)** IECs were stimulated with 20 ng/ml IFN-λ3 in the absence or presence of 750 µM VPA for 20 h (lower panel). Control cells were not treated with IFN-λ (upper panel). Cells were infected with vesicular stomatitis virus-GFP (MOI 1) for 1 h and analyzed for eGFP expression by flow cytometry 8 h post-infection. Representative FACS dot plots show the percentage of eGFP-expressing cells. Graph represents mean eGFP frequency for each condition (*n* = 3, mean ± SEM). **(D)** IECs harboring Mx2tRFP were stimulated with 20 U/ml IFN-β or 25 ng/ml IFN-λ3 in the absence or presence of the indicated concentrations of MS275. Frequency of Mx2tRFP expression was determined by flow cytometry after 24 h (*n* = 3, mean ± SEM). **P ≤ 0.01 by paired *t*-test. **(E)** IECs were stimulated with IFN in the absence or presence of the indicated concentrations of I-BET151. Percentages of Mx2tRFP-positive cells are given (*n* = 3–9, mean ± SEM). *P* values were calculated by one-way ANOVA (*P ≤ 0.05, ****P ≤ 0.0001). **(F)** IECs harboring Mx2tRFP were stimulated with 20 ng/ml IFN-λ3 in the absence or presence of 750 µm VPA. As indicated, different concentrations of I-BET151 were added during stimulation, and flow cytometry was performed after 24 h (*n* = 3, mean ± SEM). *P* values were calculated by one-way ANOVA, followed by Tukey’s Multiple Comparison Test (***P ≤ 0.001).

To test the possible influence of epigenetic alterations on the cellular response to IFNs, the effect of HDAC inhibitors on the frequency of ISG induction upon IFN stimulation was examined. As shown above, even saturating concentrations of IFN-λ3 failed to induce Mx2tRFP expression in all cells and to establish protection from VSV infection. However, addition of VPA, which inhibits class I and II HDACs (with a high potency for class I HDACs), was able to significantly increase the frequency of responding cells for all tested concentrations of IFN-λ3 (Figure 3A). This effect was confirmed for other ISGs by qRT-PCR (Figure 3B). Finally, VPA added together with IFN-λ3 elevated antiviral activity (Figure 3C). We also determined Mx2-driven Luciferase activity in the cell line IEC-Mx2Luc-10 upon HDAC inhibition. Stimulation with IFN-λ3 in the presence of VPA led to a strongly increased Mx2-Luciferase expression (Figure S3A in Supplementary Material). In contrast, VPA addition did not modulate Mx2 reporter gene activity of IFN-β to the same extent (Figure S3A in Supplementary Material). Accordingly, the frequency of Mx2tRFP expression in cells stimulated with different doses of IFN-β was not affected by the presence of VPA (Figures S3B,C in Supplementary Material). Of note, the effect of VPA was only observed when administered simultaneously with type III IFN; pretreatment of cells with VPA did not change the frequency of Mx2tRFP induction upon subsequent IFN-λ stimulation (Figure S3D in Supplementary Material). In addition, other HDAC inhibitors such as MS275 and TSA induced a similar increase in the number of Mx2tRFP-expressing cells upon IFN-λ3 stimulation but did not affect IFN-β activity (Figure 3D; Figure S3E in Supplementary Material). Together, HDAC inhibition enhances the ability of activated STATs to install ISG expression, indicating the involvement of epigenetic regulatory mechanisms in ISG gene expression upon IFN-λ stimulation.

Next, we interfered with the recruitment of readers of histone acetylation using the BRD3/4-specific inhibitor I-BET151. Inversely, the addition of I-BET151 suppressed IFN-λ3-dependent Mx2tRFP induction completely, whereas the ability of high concentrations of IFN-β to fully activate the Mx2 promoter was not impaired (Figure 3E; Figure S3A in Supplementary Material) and slightly reduced the frequency of Mx2-driven tRFP and luciferase reporter gene expression at low doses of IFN-β (20 U/ml IFN-β) (Figure 3E; Figure S3A in Supplementary Material). Thus, the threshold levels for IFN-λ-stimulated ISG expression depend on histone acetylation. Notably, VPA does not override the effect of I-BET151 (Figure 3F), confirming the dependence of BRD3/4 action on histone acetylation for ISG induction. We asked whether the effect of HDAC inhibition would be manifested on ISG promoters. Thus, IECs were stimulated with IFN-λ3 and a medium IFN-β concentration that induces Mx2tRFP expression in less than 50% of cells in the absence or presence of VPA. ChIP experiments were performed, and proximal promoter regions of three prototypical ISGs were assayed for histone H3K9 acetylation that is known to be sensitive toward treatment with VPA (26, 27). All tested ISG promoter regions showed increased levels of H3K9 acetylation upon IFN-λ3 stimulation (Figure S3F in Supplementary Material). Interestingly, histone H3K9 acetylation was slightly reduced by the addition of VPA, indicating that these sites are not the target of the HDAC inhibition effect. Despite the fact that a medium concentration of IFN-β (50 U/ml) indeed induced Mx2RFP expression to slightly higher frequencies compared to that by IFN-λ3, the level of H3K9 acetylation was not increased 5 h after stimulation.

Together, these observations suggest that IFN-λ-dependent gene induction in IECs depends mainly on histone acetylation events and that HDAC activity is a critical factor to control the threshold of promoter induction. In contrast, IFN-β-induced promoter activation is insensitive toward inhibition of histone acetylation. Thus, both pathways differ in the extent of the influence of chromatin modifications on gene induction.

### Polarization of IECs Reinforces IFN-λ Responsiveness

**Figure 4:**
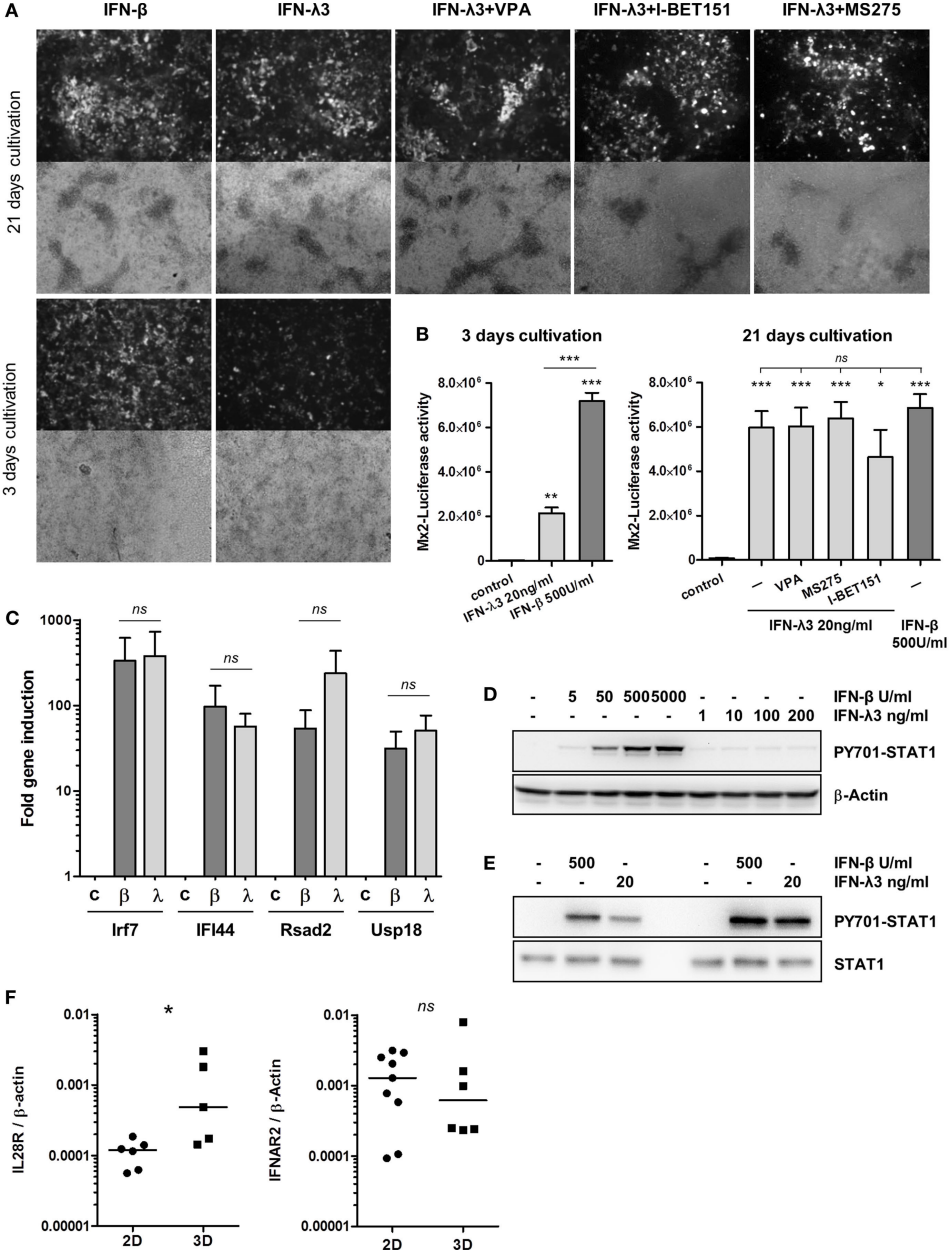
Cell polarization increases the responsiveness to interferon (IFN)-λ. **(A)** intestinal epithelial cell lines (IECs) harboring Mx2tRFP were cultured on transwell inserts for 3 and 21 days. Cells were treated with 500 U/ml IFN-β or 20 ng/ml IFN-λ3. As indicated, IFN-λ3 stimulation was done together with 750 µM valproic acid (VPA), 500 nM MS275, and 500 nM I-BET151. Mx2tRFP expression was determined 24 h after stimulation by fluorescence microscopy. Representative images are shown. Data are representative of three independent experiments. **(B)** IEC-Mx2Luc-10 cells containing the Mx2-Luciferase reporter were cultured on transwell inserts for 3 and 21 days as indicated. Cells were treated with 500 U/ml IFN-β or 20 ng/ml IFN-λ3. IFN-λ3 stimulation was done together with 750 µM VPA, 500 nM MS275, and 500 nM I-BET151 as indicated. Luciferase activity was determined 20 h after stimulation (*n* = 3, mean ± SEM). *P* values were calculated by one-way ANOVA, followed by Tukey’s Multiple Comparison Test (*P ≤ 0.05, **P ≤ 0.01, ***P ≤ 0.001). *P* values are given for differences among stimulated groups and control group (directly above columns) and between IFN-stimulated groups. **(C)** IECs cultured on transwell inserts for 21 days were treated with 500 U/ml IFN-β (β) and 20 ng/ml IFN-λ3 (λ) for 16 h. RNA was isolated, and qRT-PCR was used to determine the expression of ISGs. Fold induction after normalization to β-Actin is depicted (*n* = 3, mean ± SEM). *P* values were calculated by Mann–Whitney U test (*ns*, not significant). **(D)** IECs cultured on standard cultures dishes were treated with increasing concentrations of IFN-β and IFN-λ3 for 1 h. Western blot analysis was performed using antibodies directed against P-Y701 STAT1 and β-Actin. **(E)** IECs cultured on transwell inserts were treated with 500 U/ml IFN-β and 20 ng/ml IFN-λ3 for 1 h. Cells were lysed, and protein extracts were analyzed by Western blotting using antibodies directed against P-Y701 STAT1 and total STAT1 protein. Results of two independent experiments are shown. **(F)** IECs were cultured on standard plastic dishes (2D) or on transwell inserts (3D) for 21 days. RNA was isolated, and qRT-PCR was used to determine the expression of IL28R and IFNAR2. Receptor expression was normalized to β-Actin. *P* values were calculated by Mann–Whitney *U* test (*P ≤ 0.05).

All experiments with the immortalized epithelial cell line described above were performed on flat-bottom plastic culture dishes. Under these conditions, IFN-β responsiveness was robust, whereas only a moderate response was observed following IFN-λ stimulation. These results are in accordance with previous studies and further demonstrate that type I and type III IFN signaling realizes gene induction with different kinetics (8, 25). Importantly, a hallmark of the mature epithelium *in vivo* is an apical–basolateral cell polarization, an intrinsic feature of epithelial surface barrier formation. This phenotype can also be achieved *in vitro* by long-term cultivation on semipermeable transwell filter inserts (28). Under these conditions, intestinal epithelial cells polarize into apical and basolateral membrane domains with tight cell junctions and 100% confluency (21). To analyze the influence of epithelial polarization on IFN signaling, IECs were routinely grown for at least 21 days on transwell inserts. TEER was measured to confirm cellular polarization and confluency (Figure S4A in Supplementary Material). For comparison, cells were cultured for only 3 days on transwell inserts resulting in incomplete polarization. Cells were stimulated for 20 h with either type I or type III IFN, and Mx2tRFP expression was analyzed by fluorescence microscopy. Stimulation of fully polarized confluent enterocytes with IFN-λ3 in contrast to conventionally cultured (Figure 1) or short-term transwell-cultured cells reached a similar signal strength as that with IFN-β with respect to both the number of activated cells and the intensity of cellular Mx2tRFP expression (Figure 4A). Further, the HDAC inhibitors VPA and MS275 as well as the addition of I-BET151 did not alter the IFN-λ-induced Mx2tRFP expression as observed under conventional culture conditions (Figure 4A). To quantify the results obtained by microscopic analysis of Mx2tRFP induction, we determined IFN-λ3-induced Mx2-Luciferase activity in the cell line IEC-Mx2Luc-10. Luciferase measurements indicated that IFN-β-mediated induction of the Mx2 promoter did not change upon polarization (Figure 4B). However, IFN-λ activity strongly increased after 21 days of cultivation on Transwell inserts, and HDAC inhibition did not further stimulate Mx2-driven Luciferase expression (Figure 4B). Thus, these quantitative data confirm the results obtained from Mx2tRFP expression. Of note, the IFN-λ-mediated induction of other ISGs such as IRF7, IFI44, Rsad2, and USP18 in fully polarized and confluent cells grown on transwell inserts reached levels similar to those with IFN-β exposure (Figure 4C).

IFN-stimulated gene and Mx2 reporter gene induction and antiviral activity depend on STAT1 activation. Western blot analysis revealed that IFN-λ3 induced a much weaker STAT1 phosphorylation at tyrosine residue 701 under conventional culture conditions compared to that by IFN-β (Figure 4D; Figures S4B,C in Supplementary Material). In contrast, IFN-λ3 stimulation of transwell-grown polarized cells resulted in an enhanced Y701-phosphorylation of STAT1 reaching levels comparable to levels obtained after IFN-β treatment (Figure 4E). In order to understand the source of the increased type III IFN responsiveness in polarized IECs, we compared gene expression of the IFN-λ receptor between IECs cultivated under non-polarizing versus polarizing conditions. Interestingly, IECs displayed elevated levels of IL28R mRNA when cellular polarization was established, whereas the expression of the type I receptor chain IFNAR2 was not affected (Figure 4F).

**Figure 5:**
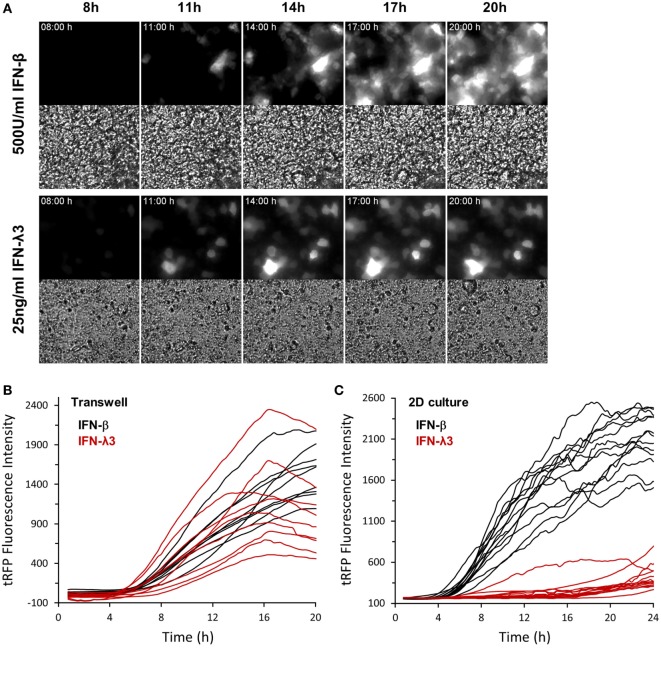
Cell polarization abrogates differential expression kinetics of type I and type III interferons (IFNs). Intestinal epithelial cell lines (IECs) harboring Mx2tRFP were cultured on transwell inserts for 21 days. Cells were treated with 500 U/ml IFN-β and 25 ng/ml IFN-λ3. Time-lapse fluorescence microscopy was used to follow the induction of Mx2tRFP in live cells. **(A)** Representative fluorescence and corresponding bright field images at selected time points are shown. **(B)** Mx2tRFP fluorescence intensities were quantified using ImageJ software. **(C)** IECs grown on standard culture dishes (2D) were treated with 500 U/ml IFN-β and 25 ng/ml IFN-λ3 and subjected to time-lapse fluorescence microscopy. Mx2tRFP fluorescence intensities were quantified over time using ImageJ software.

Next, we tested whether IEC polarization also exerts a significant influence on the described delay in ISG expression following IFN-λ3 stimulation (compare Figure S1A in Supplementary Material). IECs harboring Mx2tRFP were grown for 21 days on transwell inserts and stimulated with both types of IFNs. Time-lapse microscopy revealed that IFN-λ3 stimulation of fully polarized cells indeed resulted in a rapid induction of Mx2tRFP expression with onset times of 5–7 h similar to what was observed after IFN-β exposure (Figures 5A,B). In contrast and consistent with our previous results, IFN-λ-induced Mx2tRFP expression under standard 2D culture conditions exhibited delayed onset time points after IFN-λ3 stimulation varying between 6 and 14 h after stimulation (Figure 5C). Here, IFN-λ3-induced fluorescence intensities did not reach levels observed after administration of high concentrations of IFN-β. Of note, VPA co-treatment did not alter the kinetics of Mx2tRFP gene induction upon IFN-λ stimulation under standard 2D culture conditions (Figure S5A in Supplementary Material). Together, these findings indicate that epithelial polarization abolishes the differences between type I and III IFN signaling and specifically enhances IFN-λ sensitivity.

### Efficient Response to IFN-λ in Small Intestinal Organoids

**Figure 6:**
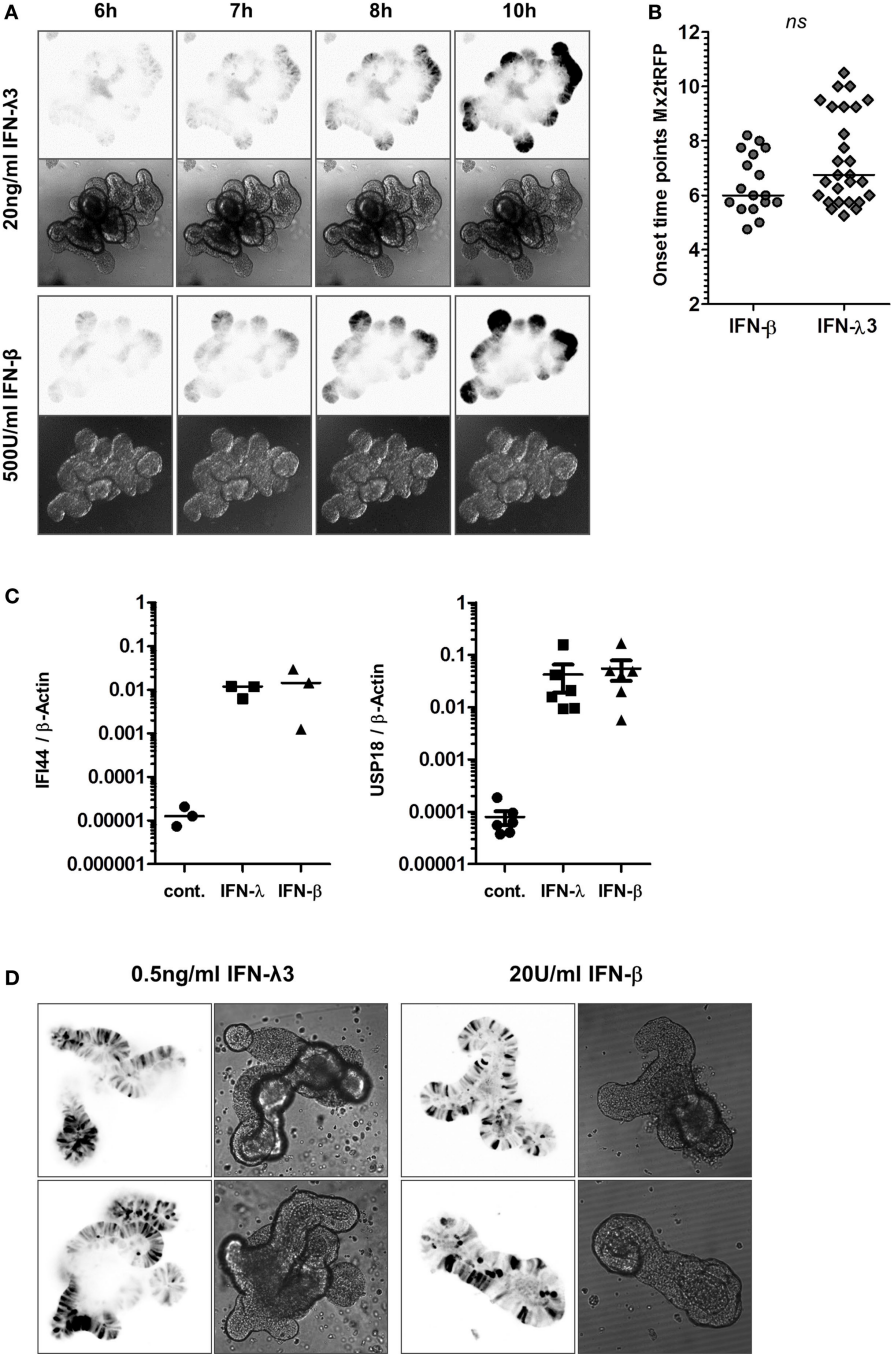

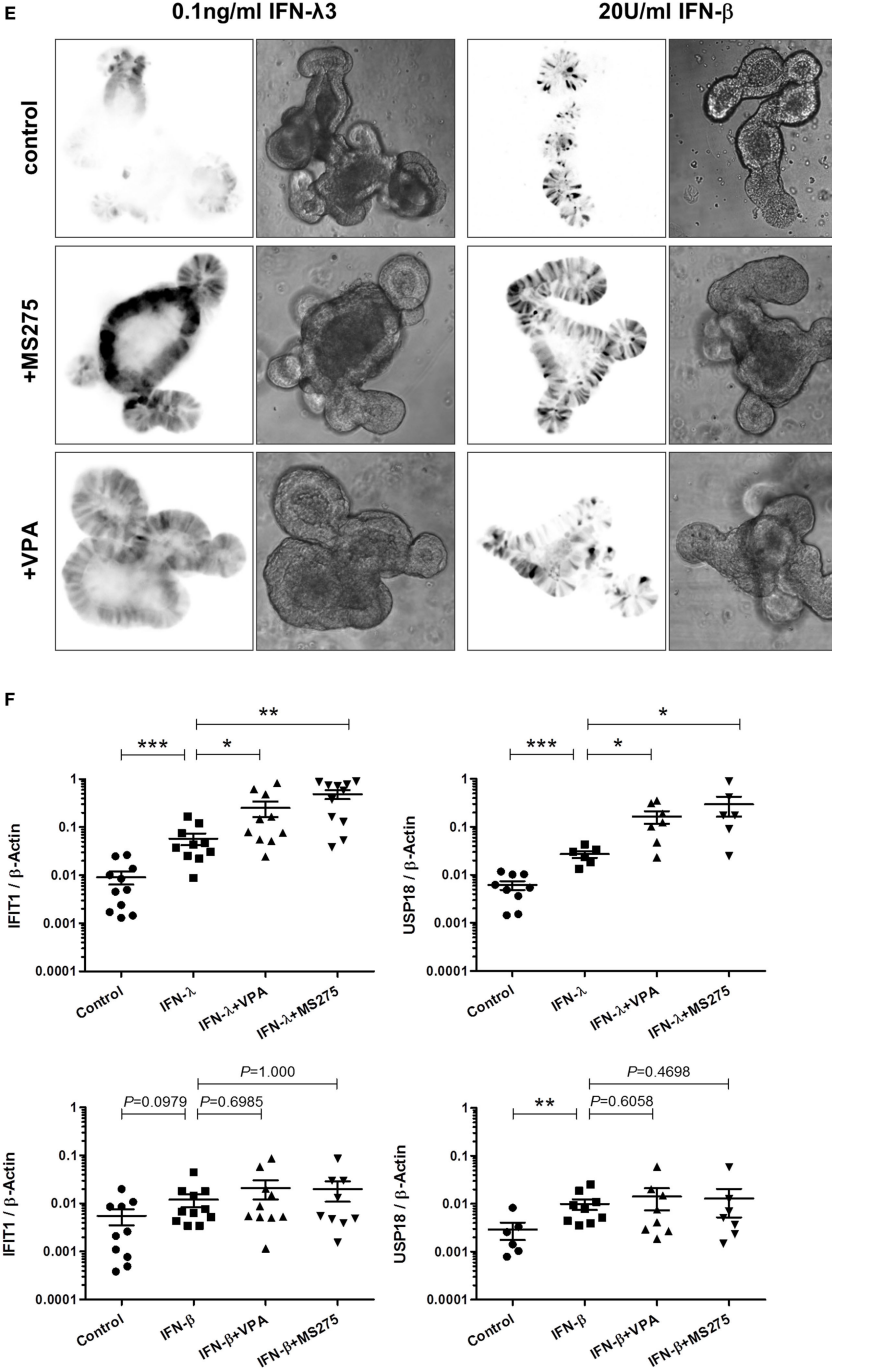
High responsiveness to interferon (IFN)-λ in intestinal organoids. Murine small intestinal crypts were isolated from Mx2tRFP transgenic mice. Mature organoids were obtained after incubating small intestinal crypts for 9–10 days in Matrigel. **(A)** Mx2tRFP organoids were treated with 20 ng/ml IFN-λ3 or 500 U/ml IFN-β and subjected to time-lapse confocal microscopy. Optical sections were acquired using identical acquisition settings for both types of IFNs. Mx2tRFP expression is shown at selected time points. Fluorescent images were inverted using ImageJ software. **(B)** Onset time points of Mx2tRFP expression were determined from time series after IFN-β and IFN-λ3 stimulation. *P* value was calculated by Mann–Whitney *U* test; *ns*, not significant. **(C)** Mx2tRFP organoids were treated with 20 ng/ml IFN-λ2 and 500 U/ml IFN-β for 9 h. RNA was isolated, and qRT-PCR was used to determine the expression of IFI44 and USP18. IFN-stimulated gene expression was normalized to β-Actin. **(D)** Mx2tRFP organoids were treated with 0.5 ng/ml IFN-λ3 and 20 U/ml IFN-β for 20 h and subjected to confocal fluorescence microscopy. For both IFN treatments, two representative single plane images show Mx2tRFP expression from intact organoids. Fluorescent images were inverted using ImageJ software. **(E)** Mx2tRFP organoids were treated for 16 h with 0.1 ng/ml IFN-λ3 or 20 U/ml IFN-β in the absence or presence of 750 µM valproic acid (VPA) and 0.51 µM MS275. Confocal fluorescence microscopy was used to collect single plane images from intact organoids 20 h after stimulation. Optical sections were acquired using identical acquisition settings for each type of IFN. Gain and offset were adjusted to use the entire dynamic range of the detector and to avoid saturation of the tRFP signal. Fluorescent images have been inverted using ImageJ software, and representative images showing Mx2tRFP expression are presented. **(F)** Mx2tRFP organoids were treated with 0.1 ng/ml IFN-λ3 and 20 U/ml IFN-β in the absence or presence of 750 µM VPA and 0.51 µM MS275. mRNA levels for IFIT1 and USP18 were determined by quantitative real-time PCR and normalized to β-Actin. *P* values were calculated by Mann–Whitney U test (*P ≤ 0.05, **P ≤ 0.01, ***P ≤ 0.001).

Recent studies had indicated that stem cell-derived small intestinal epithelial organoid cultures recapitulate the polarization and differentiation observed in the adult intestine *in vivo* (22, 29). These organoid structures are characterized by a crypt-villus organization, epithelial polarization, and a functional lumen. To determine the characteristics of gene induction in response to type I and type III IFNs in gut organoid cultures, we made use of a transgenic mouse line harboring the Mx2tRFP reporter. Intestinal organoids established from Mx2tRFP transgenic reporter mice were treated with IFN-λ3 or IFN-β, and the kinetics of Mx2tRFP induction were determined by time-lapse confocal microscopy (Figure 6A). Upon stimulation with high dose of IFN-λ3 (20 ng/ml) or IFN-β (500 U/ml), we found no marked differences regarding the onset time points for tRFP expression (Figure 6B). Analysis of the mRNA induction of the ISGs IFI44 and USP18 by quantitative real-time PCR indicated an equal activity of both types of IFNs in the stem cell organoid cultures (Figure 6C). Interestingly, a scattered heterogeneous responsiveness of the epithelial cells within the analyzed organoids was observed upon administration of low concentrations of both types of IFNs (Figure 6D). Here, a high cell-to-cell variability in gene induction with distinct Mx2 expressing and non-expressing cells was detected. This heterogeneity could be reduced by addition of the HDAC inhibitors VPA and MS275, enhancing the fraction of IFN-λ-reactive cells as well as the overall reporter gene expression level (Figure 6E). In accordance with the results obtained in the 2D and transwell cultivation system, HDAC inhibition did not alter the variability of Mx2tRFP expression toward low concentrations of IFN-β (Figure 6E). Quantitative mRNA analysis of the prototypical ISGs IFIT1 and USP18 confirmed that IFN-λ activity but not that of IFN-β is enhanced under conditions of diminished HDAC activity (Figure 6F).

Overall, the presented data of the stem cell organoid system indicated that intestinal epithelial cells *in situ* are fully responsive to IFN-λ and that this response is comparable to the response of immortalized epithelial cells cultured under fully polarizing conditions. Moreover, the physiological relevant stem cell organoid system underlined the divergent role of HDAC activity as a restriction factor for type III but not type I IFN signaling.

## Discussion

In this report, we took advantage of the fact that an immortalized IEC and intestinal stem cell organoids react to types I and III IFNs. These cellular models were used in combination with genetic luciferase and fluorescent reporter constructs that could reflect the global ISG response (30) and the resulting antiviral status (Figure 2A). This approach allowed us to analyze the quantitative response over time both for the cell population and on the single cell level. Our analysis for the first time revealed that the difference in the quantitative response to both IFNs is largely based on bimodal decisions of cells, i.e., a yes/no decision of each individual cell upon stimulus exposure. This finding confirms earlier published results obtained from a different cell model (19). It could also be observed in stem cell organoids and may thus represent the behavior of intestinal epithelial cells *in vivo*. Organoids better resemble intact mature intestinal epithelial layers in their cellular composition and function but still allow to examine individual living cells under defined conditions. However, the *in vivo* situation cannot be directly compared with cell culture systems since constitutive type I and III IFN expression (31, 32) and activities from other cell types might prime the cellular response toward IFN or mask the effects of the stochastic cellular response. In addition to the bimodality, we demonstrate that IFN stimuli modulate the strength of reporter gene expression within the responding cells as represented by the MFI values in tRFP reporter cells (Figure 1). This effect, however, appears to be inferior to the bimodality of the response within a cell population. Moreover, it is mainly restricted to the action of type I IFNs, thus representing one of the differences between the cellular responses to both IFN types.

The discovery that type I and type III IFNs triggered the same Jak-STAT signaling pathway supported the idea that both types of IFNs would have identical functions. Indeed, both cytokines were reported to induce comparable patterns of gene expression and similar biological effects (2, 7, 8). The differential tissue distribution of the respective receptor molecules led to the concept that type III IFNs act on specific cell types, whereas type I IFNs affect all nucleated cells in the body. Our results challenge this view since we could find significant differences in the kinetics of ISG expression, the heterogeneity of the responding cell population, and cellular as well as epigenetic requirements for type I and III IFN-mediated antiviral activities.

Our results define several parameters that ultimately determine the cellular responsiveness to IFNs: IFN concentration, epigenetic modulation, and polarization status of the cells. First, the concentration of both IFNs plays a key role. Type I IFN at high concentrations is able to achieve a nearly completely homogenously reacting cell population. In contrast, a low responsiveness in combination with a delayed kinetic characterize the IFN-λ response in several cell models (8, 25, 33–35). Our results using epithelial cells cultured under conventional non-polarizing conditions confirm these observations and extend them by showing that only a fraction of cells is responding, even at high concentrations of IFN-λ. This is associated with a weak phosphorylation of STAT1.

In stark contrast, cultivation under polarizing conditions results in high IFN-λ responsiveness associated with a high fraction of responding cells within a population and strong STAT1 phosphorylation. Thus, under polarization conditions, the responsiveness to IFN-λ is largely comparable to that of IFN-β. A straightforward explanation is given by the fact that the expression of the IFN-λ receptor is, in contrast to the type I IFN receptor, dependent on the polarization status of the cells (Figure 4F). Polarization leads to a higher expression of the IFN-λ receptor and a higher extent of STAT phosphorylation. This reflects IFN-β activity in polarized and non-polarized cells. The role of polarization is confirmed in the organoid culture system where a closely related read-out for IFN-β and IFN-λ was found (Figures 6A,C). However, administration of low concentrations of both types of IFNs resulted in high cell-to-cell variability in Mx2tRFP induction (Figure 6D).

Another difference between the responses to the two types of IFNs concerns the modulation of the epigenetic status with HDAC blockers. While type I IFN activity in all conditions of IEC cultivation and in organoids is completely insensitive to HDAC inhibition, IFN-λ responsiveness is strongly increased. This is true for the intestinal epithelial cells in the non-polarized status and in organoids. Interestingly, the increase in Mx2 expression in IECs depends exclusively on the number of responding cells and not on the expression strength per cell. Since the HDAC inhibitor-mediated enhancement of the IFN-λ response is reduced by I-BET151 administration, histones seem to be the functionally relevant target of the HDAC inhibitors. Several reports indicate that the acetylation level of H3K9 increases across the genome following VPA treatment (26, 27). However, the global change in H3K9 acetylation and other histone modifications induced by HDAC inhibition are not recapitulated at all individual promoter sites as measured by ChIP (36, 37). We show here that IFN-λ3 stimulation results in elevated levels of H3K9ac at proximal promoter regions when compared to those in untreated cells. However, the increased responsiveness of IECs by VPA co-treatment is not straightforward since HDAC inhibition is not reflected in a further increase in H3K9 acetylation at the analyzed proximal promoter regions (but rather limits its elevation) (Figure S3F in Supplementary Material). We propose that HDAC inhibition targets remote promoter elements or control regions that are necessary to induce expression of a larger cluster of ISGs. However, we assume that IFN-λ stimulation of cells with reduced HDAC activity mediates high level of histone acetylation at such sites providing a chromatin context that allows the expression of ISGs in an otherwise unresponsive cell population. This distinguishes this scenario from the epigenetic reactions induced by IFN-β. Further, the complexity of epigenetic regulation during IFN-mediated ISG induction and the temporal influence of HDAC inhibition on different histone modifications still have to be determined.

In contrast to IFN-λ stimulation, ChIP analysis indicated that H3K9 acetylation was not altered during IFN-β-mediated ISG promoter activation (Figure S3F in Supplementary Material; 50 U/ml IFN-β), suggesting that H3K9 acetylation at these sites is not important for gene induction. This is in line with the observation that VPA-mediated reduction of HDAC activity does not affect submaximal Mx2tRFP and ISG induction upon IFN-β stimulation. Further, addition of the BRD3/4 inhibitor I-BET151 did not reduce gene expression at IFN-β concentrations higher than 50 U/ml (Figure 3E; Figure S3A in Supplementary Material). Thus, we suggest that type I and type III IFN signaling induces different spectra of activating histone modifications at target genes in epithelial cells. In this scenario, other histone modifications than H3K9 acetylation possess an overriding importance for IFN-β. Indeed, active histone marks such as H3K4 and H3K79 trimethylation in the promoter regions of ISGs were found to be induced upon type I IFN treatment (38, 39). However, at low doses of IFN-β, the recruitment of readers of histone acetylation is more relevant, since I-BET151 addition leads to a distinct reduction in Mx2-Luciferase activity (Figure 3E; Figure S3A in Supplementary Material, 20 U/ml IFN-β). Since ISGs are targets for both types of IFNs and the HDAC-modified chromatin context is only relevant for IFN-λ, a higher complexity of signaling for IFN-β under non-polarized conditions has to be assumed. Currently, biochemical evidence for such a difference is not yet available.

An important aspect concerns the fact that the differential responsiveness toward IFN-λ is reflected by the percentage of responding cells. This suggests that the chromatin status (histone code) defines the probability for responsiveness. The responsiveness toward IFN-β is also bimodal and concentration-dependent, but is modulated neither by HDAC inhibition nor by polarization. Assuming that this effect is also based on the chromatin status, other types of histone modifications have to be considered. Binary responses have been shown to be evoked by positive feedback loops based on autocatalytic switches (40, 41). It will be of interest to see if such mechanisms apply for the bimodal response to IFNs and which molecular basis is underlying the probabilistic gene induction for both types of IFNs.

The biological function of the reported bimodality in contrast to graded induction of other genes is unknown. We speculate that it may be of advantage to maintain individual unprotected cells in an organism upon exposure to low levels of IFNs. This could allow limited virus propagation and thereby stimulation and priming of the adaptive immune system to provide subsequent protection. Alternatively, IFN-responding cells might alter their physiology in a way that hinders critical physiological functions of the gut epithelium *in vivo*. Therefore, it might be of advantage to maintain the full functionality of at least a fraction of enterocytes.

Our results ascribe a special role to IFN-λ in comparison to type I IFNs that goes beyond cell type specificity. Its transcriptional activity is strongly influenced by cell polarization and underlies a bimodal decision process and epigenetic modifications further expanding our knowledge on the complex regulation of the intestinal epithelial response to type I and type III IFNs.

## Author Contributions

SB, MW, HH, and MK designed the study. SB and MW performed most of the experiments. TS, LK, and MK performed some experiments. SB, MW, LK, and MK analyzed the data. DW provided essential tools. HH, MH, and MK wrote the article. SB and MW contributed equally to this work.

## Conflict of Interest Statement

The authors declare that the research was conducted in the absence of any commercial or financial relationships that could be construed as a potential conflict of interest.

## Supplementary Material

Video S1 Kinetics of Mx2tRFP induction in intestinal epithelial cell lines (IECs) grown under standard culture conditions. Time-lapse imaging of IECs Mx2tRFP stimulated with 500 U/ml interferon (IFN)-β (above) and 25 ng/ml IFN-λ3 (below).

Video S2 Kinetics of Mx2tRFP induction in polarized intestinal epithelial cell lines (IECs). IECs harboring Mx2tRFP were cultured on transwell inserts for 21 days. Time-lapse imaging of cells stimulated with 500 U/ml interferon (IFN)-β (above) and 20 ng/ml IFN-λ3 (below).
