# Supplementary material for: Cell Polarization and Epigenetic Status Shape the Heterogeneous Response to Type III Interferons in Intestinal Epithelial Cells
Source: Front Immunol. 2017 Jun 12;8:671. doi: 10.3389/fimmu.2017.00671 (PMC5467006; doi:10.3389/fimmu.2017.00671)
Supplement: Supplementary file 1 [file presentation_1.pdf]

Supplementary Material

Cell Polarization and Epigenetic Status Shape the Heterogeneous Response to Type III Interferons in Intestinal Epithelial Cells

Sudeep Bhushal, Markus Wolfsmüller, Tharini A Selvakumar, Lucas Kemper, Dagmar Wirth, Mathias W Hornef, Hansjörg Hauser, Mario Köster

Figure S1

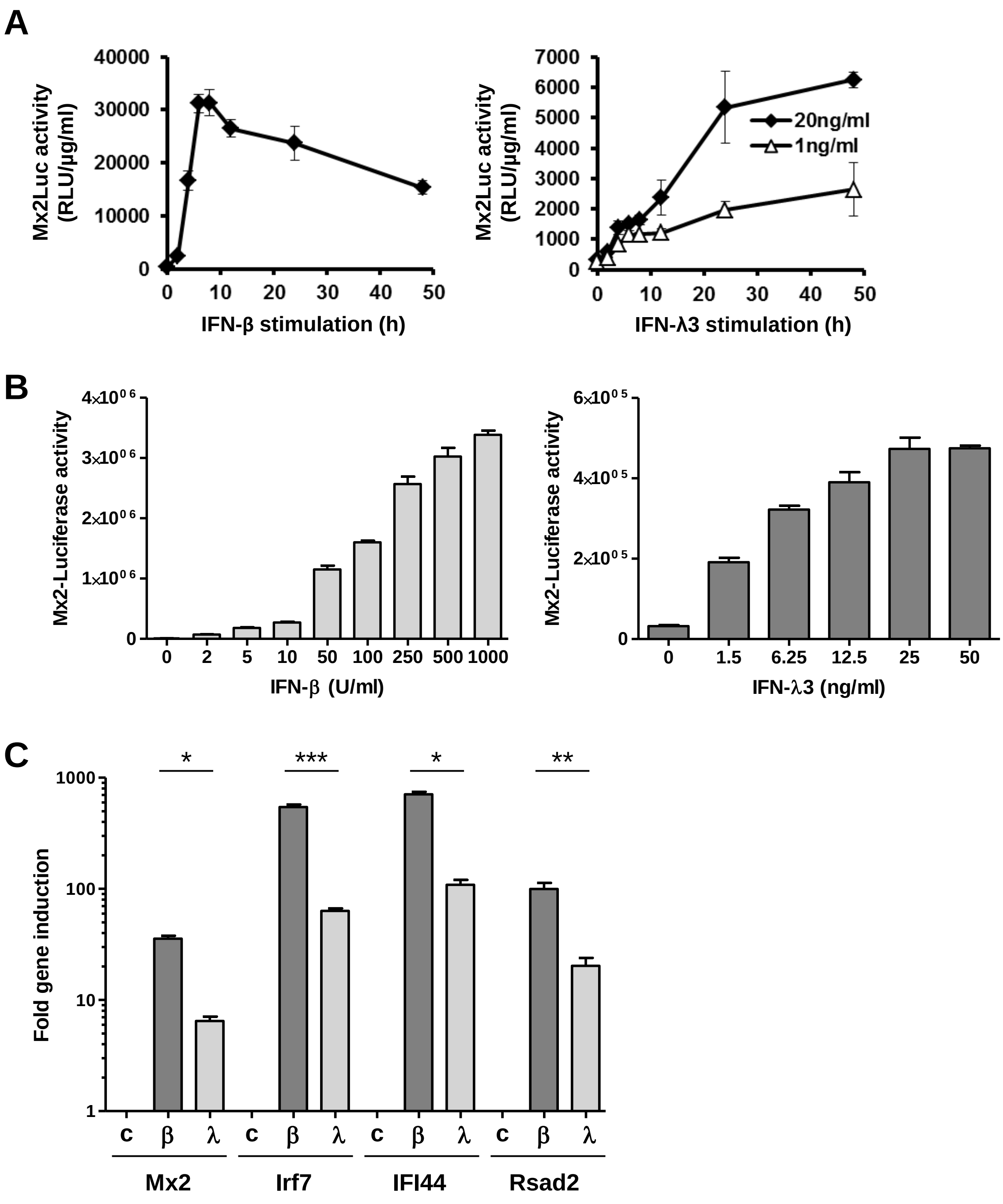

A) Intestinal epithelial cells (IECs) harboring the BAC construct Mx2Luc were stimulated with 500U/ml IFN-β, 1ng/ml IFN-λ3 and 20ng/ml IFN-λ3. Luciferase activity was determined at the indicated time points (n = 3, mean ± SEM).
B) IECs harboring Mx2Luc were stimulated with increasing concentrations of IFN-β and IFN-λ3. Luciferase activity was determined 20 hours after stimulation (n = 3, mean ± SEM).
C) IECs harboring the BAC construct Mx2tRFP were either untreated (c) or treated with 500U/ml IFN-β (β) and 20ng/ml IFN-λ3 (λ) for 16 hours. RNA was isolated and qRT-PCR was used to determine the expression of Mx2, IRF7, IFI44 and Rsad2. ISG expression was normalized to β-Actin (n = 3, mean ± SEM). *P* values were calculated by Mann-Whitney's U test (**P* ≤ 0.05, ***P* ≤ 0.01, ****P* ≤ 0.001).

Figure S1

D

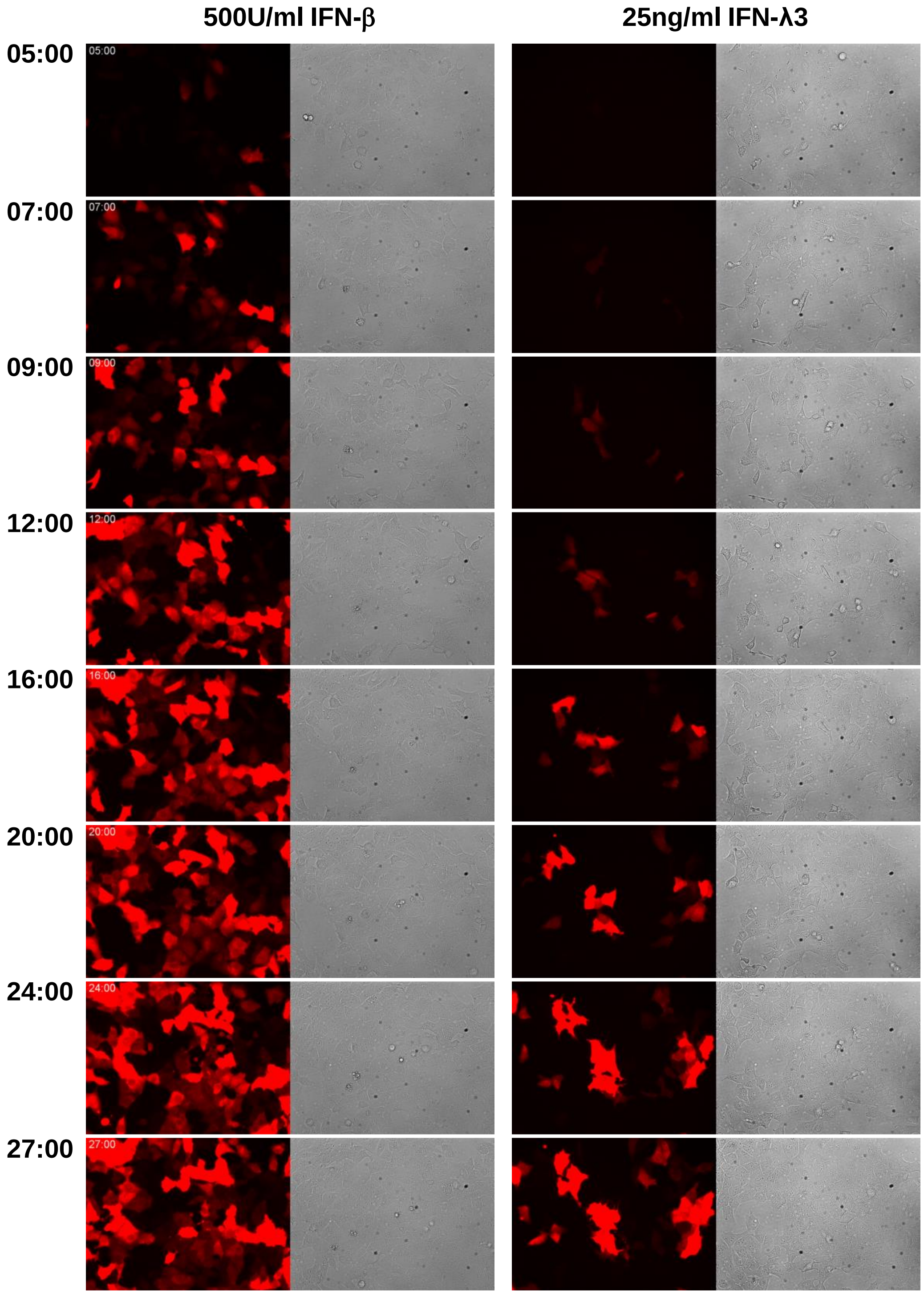

D) IECs harboring Mx2tRFP were stimulated with 500U/ml IFN-β and 25ng/ml IFN-λ3 and subjected to time-lapse fluorescence microscopy. Representative fluorescent and corresponding bright field images at selected time points are shown.

Figure S2

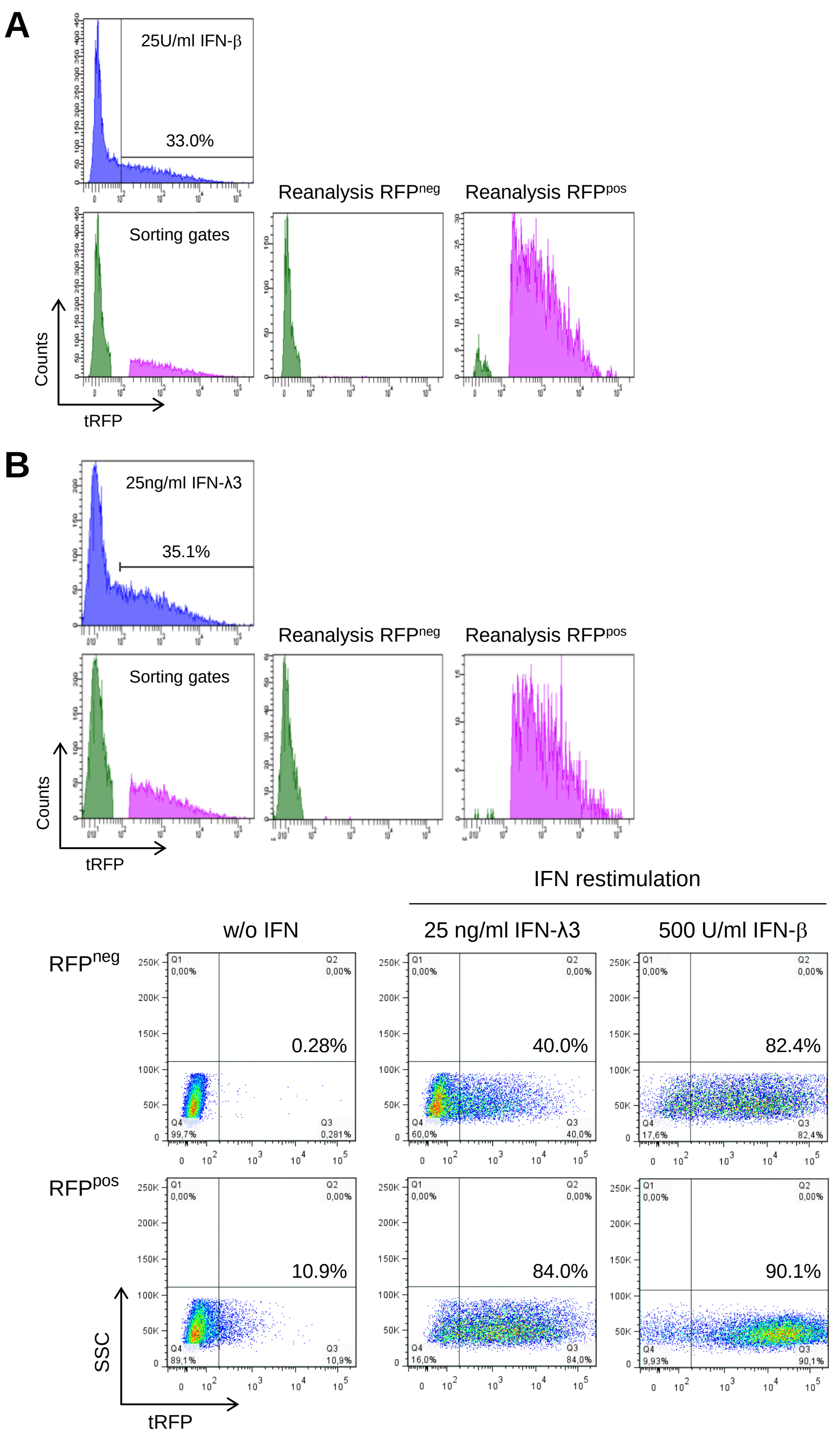

A) Reanalysis of the sorted Mx2tRFP positive and negative populations related to Figure 2B is shown.

B) IECs were stimulated for 24 hours with 25ng/ml IFN-λ3 and Mx2tRFP positive and negative populations were separated by cell sorting. Reanalysis of the sorted populations is shown. Cells were cultivated for 40 hours in the absence of IFN and responder as well as non-responder populations were restimulated for 24 hours with the indicated concentrations of IFN-β and IFN-λ3. Mx2-driven tRFP expression was measured by flow cytometry. Representative FACS dot plots are shown.

Figure S3

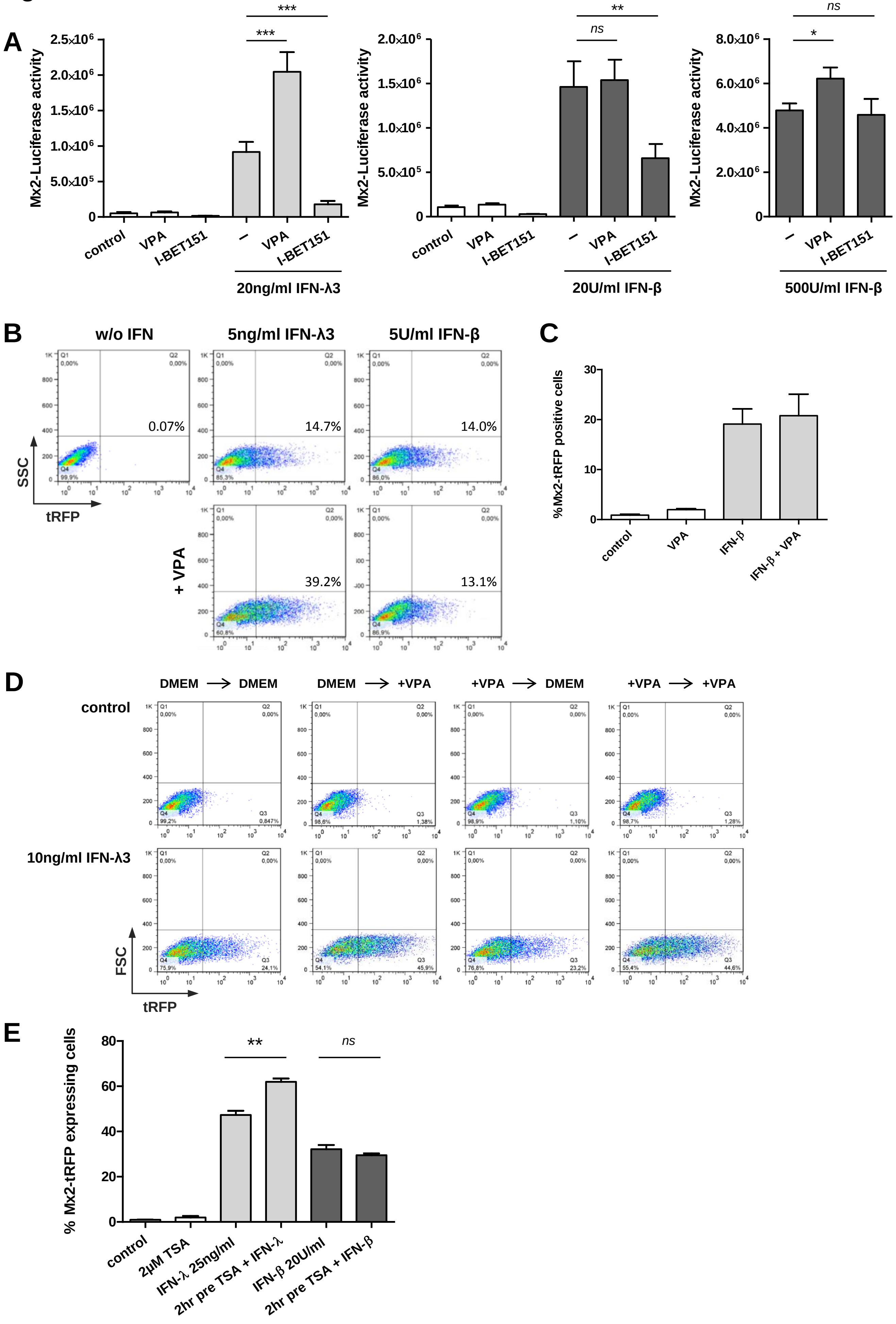
A
Mx2-Luciferase activity
control
VPA
I-BET151
20ng/ml IFN-λ3
20U/ml IFN-β
500U/ml IFN-β
***
**
ns
*
B
w/o IFN
5ng/ml IFN-λ3
5U/ml IFN-β
0.07%
14.7%
14.0%
39.2%
13.1%
+ VPA
SSC
tRFP
C
%Mx2-tRFP positive cells
control
VPA
IFN-β
IFN-β + VPA
D
DMEM → DMEM
DMEM → +VPA
+VPA → DMEM
+VPA → +VPA
control
10ng/ml IFN-λ3
FSC
tRFP
E
% Mx2-tRFP expressing cells
control
2µM TSA
IFN-λ 25ng/ml
2hr pre TSA + IFN-λ
IFN-β 20U/ml
2hr pre TSA + IFN-β
**
ns

Figure S3

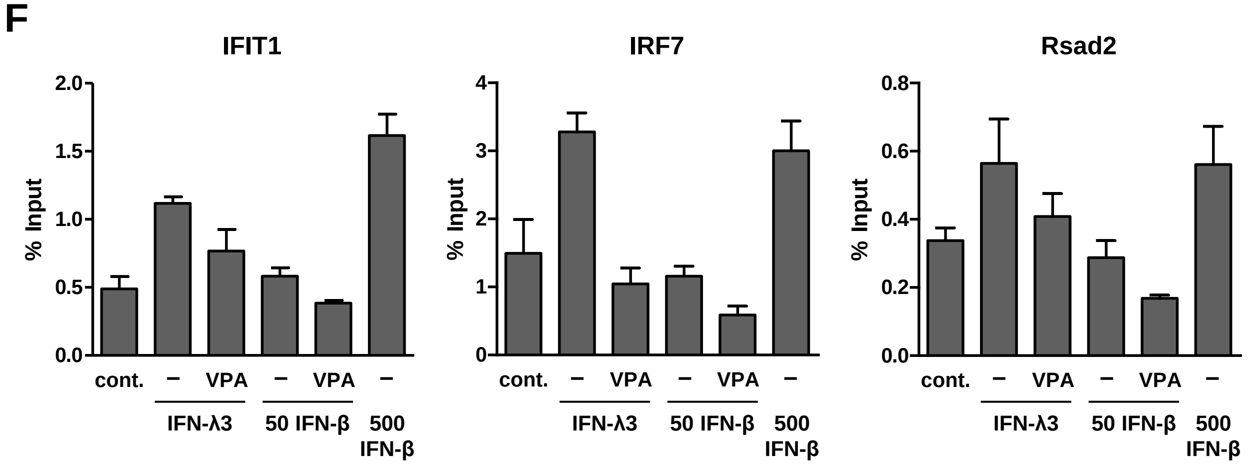

Figure S3

A) IEC-Mx2Luc-10 cells containing the Mx2-Luciferase reporter were treated with 20ng/ml IFN-λ3, 20U/ml IFN-β or 500U/ml IFN-β. Stimulation was done together with 750µM VPA or 500nM I-BET151 as indicated. Luciferase activity was determined 20 hours after stimulation (n = 9, mean ± SEM). *P* values were calculated by one-way ANOVA followed by Tukey's Multiple Comparison Test (**P* ≤ 0.05; ***P* ≤ 0.01; ****P* ≤ 0.001; *ns*, not significant).

B) IECs harboring Mx2tRFP were stimulated with 5ng/ml IFN-λ3 and 5U/ml IFN-β in the absence or presence of 750µM VPA. Frequency of Mx2tRFP expression was determined by flow cytometry after 24 hours. Representative FACS dot plots show percentage of tRFP expressing cells.

C) IECs harboring Mx2tRFP were stimulated with 10U/ml IFN-β in the absence or presence of 750µM VPA. Frequency of Mx2tRFP expression was determined by flow cytometry after 20 hours (n = 3, mean ± SEM).

D) IECs harboring Mx2tRFP were cultured for 18 hours in the absence (DMEM) or presence of 750µM VPA (+VPA). Subsequently, cells were washed and both condition were further cultivated in media without (DMEM) and with 750µM VPA (+VPA) in the absence or presence of 10ng/ml IFN-λ3. Frequency of Mx2tRFP expression was determined by flow cytometry after 20 hours. Representative FACS dot plots show percentage of tRFP expressing cells.

E) IECs harboring Mx2tRFP were cultivated for 2 hours in the absence or presence of 2µM Trichostatin A (TSA). Subsequently, cells were washed and both condition were further cultivated in media without and with 25ng/ml IFN-λ3 or 20U/ml IFN-β for 24 hours (without TSA). Frequency of Mx2tRFP expression was determined by flow cytometry (n = 3, mean ± SEM). *P* values were calculated by one-way ANOVA followed by Tukey's Multiple Comparison Test (***P* ≤ 0.01; *ns*, not significant).

F) IECs harboring Mx2tRFP were treated with 25ng/ml IFN-λ3, 50U/ml IFN-β or 500U/ml IFN-β for 5 hours. VPA (750µM) was added to the stimulation as indicated. ChIP assays were performed with H3K9ac antibody, and DNA was analyzed by Real-time quantitative PCR using primers specific for IFIT1, IRF7 and Rsad2 proximal promoter regions. Percent of input DNA was determined by comparing cycle threshold value (C_T) obtained with immunoprecipitated DNA and C_T value obtained from input DNA. Histograms are means ± SD of data from two independent pull-down experiments.

Figure S4

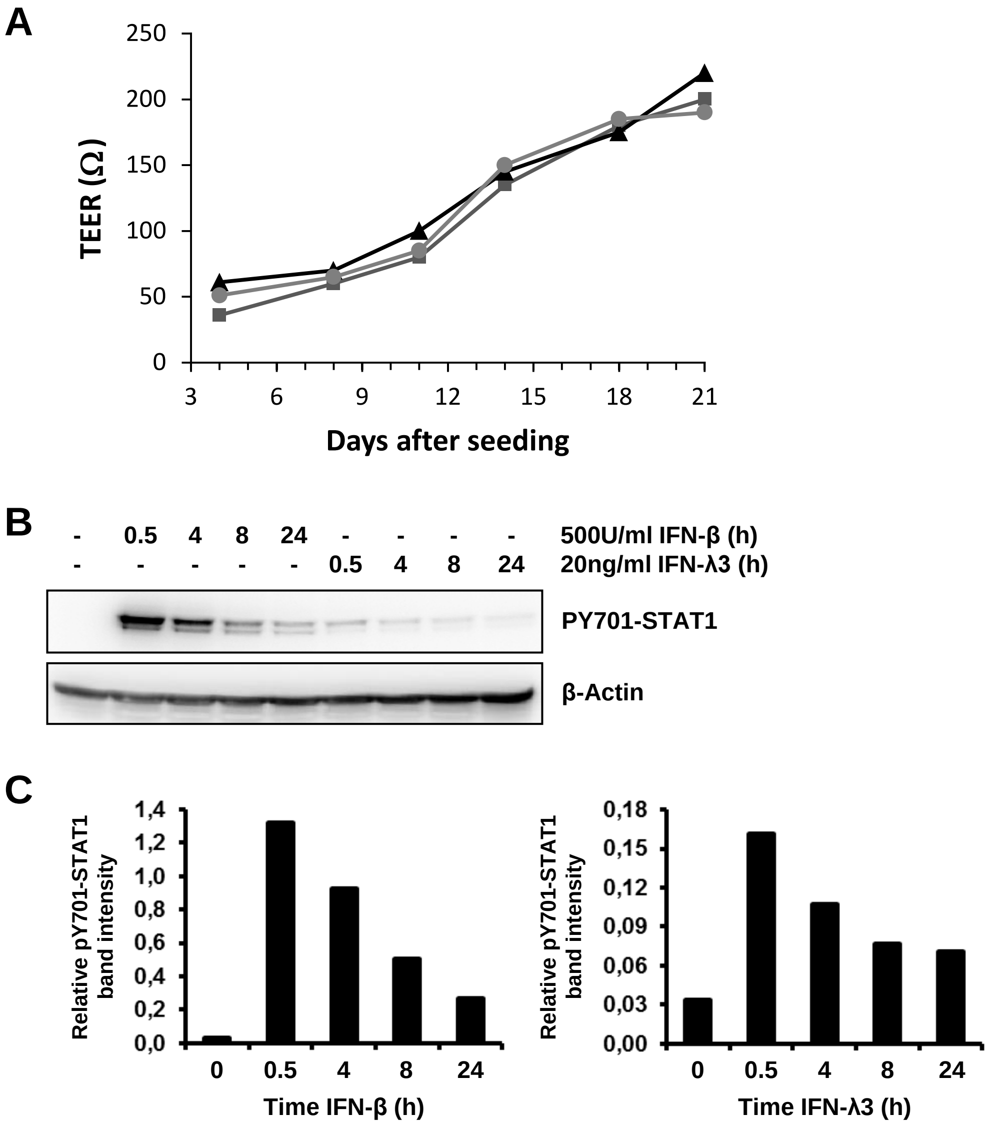

A) IECs harboring Mx2tRFP were seeded on porous cell culture inserts with a 0.4 µm pore size. Transepithelial electrical resistance (TEER) was monitored over time in triplicates and is expressed as resistance in ohms multiplied by the area of the cell culture insert (Ω*cm^2).
B) IECs cultured on standard cultures dishes were treated with 500U/ml IFN-β and 20ng/ml IFN-λ3 for the indicated periods of time. Western blot analysis was performed using antibodies directed against PY701-STAT1 and β-Actin.
C) Protein abundance of STAT1 phosphorylated at position Y701 and β-Actin were quantified. Data are presented as a ratio of PY701-STAT1 to β-Actin.

Figure S5

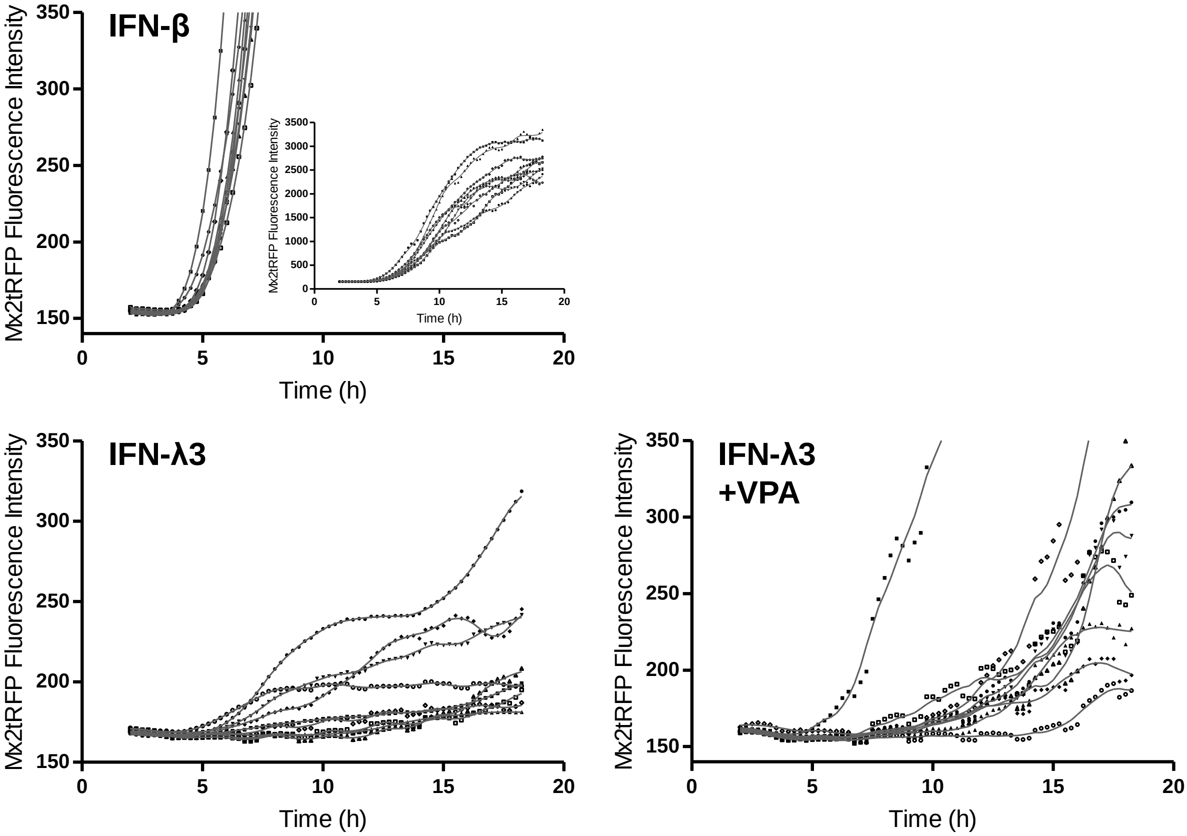

IECs harboring Mx2tRFP were treated with 500U/ml IFN-β, 20ng/ml IFN-λ3 and 20ng/ml IFN-λ3 plus 750µM VPA. Subsequently, cells were subjected to time-lapse fluorescence microscopy. Mx2tRFP fluorescence intensities were quantified in selected areas over time using ImageJ software. Curve smoothing was performed using GraphPad software related to the method of Savistsky and Golay (A. Savitzky and M.J.E. Golay, (1964). Smoothing and Differentiation of Data by Simplified Least Squares Procedures. Analytical Chemistry 36 (8): 1627-1639). Dots represent fluorescence measurements. The small insert in the upper left graph represents Mx2tRFP fluorescence intensities with enlarged x-axis.

Figure S6

A

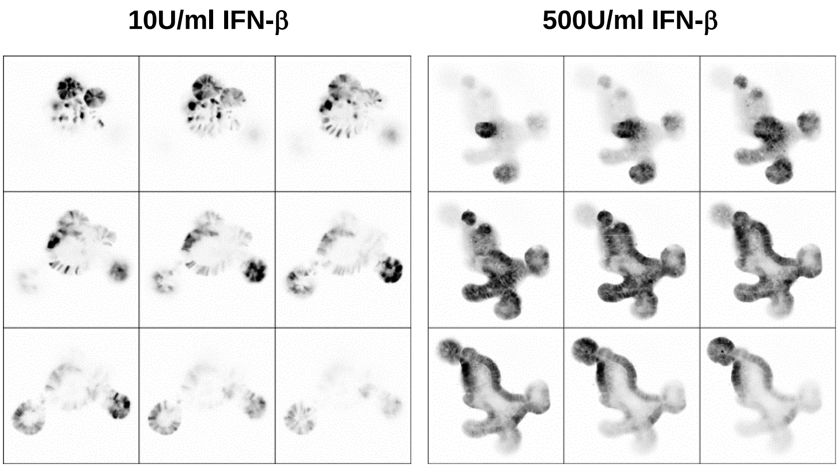

B

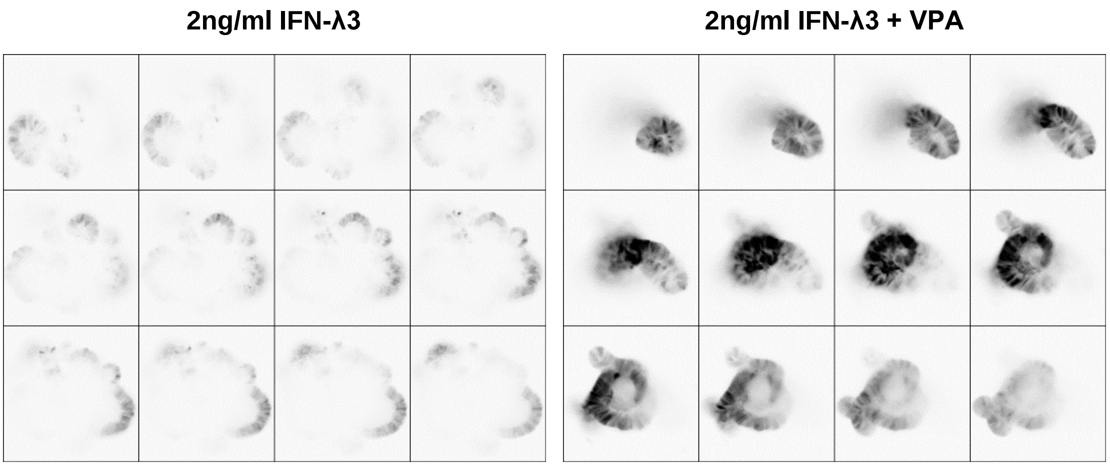

A) Murine small intestinal crypts were isolated from Mx2tRFP transgenic mice. Mature organoids were obtained after incubating small intestinal crypts for 9-10 days in Matrigel. Mx2tRFP organoids were treated with 10U/ml or 500U/ml IFN-β for 20 hours. Optical sections were acquired using confocal laser-scanning microscopy. Representative images stacks show Mx2tRFP expression from intact organoids. Fluorescent images were inverted using ImageJ software.

B) Mx2tRFP organoids were treated with 2ng/ml IFN-λ3 in the absence or presence of 750µM VPA for 20 hours and subjected to confocal laser-scanning microscopy. Optical sections were acquired using identical acquisition settings for both conditions. Gain and offset were adjusted to use the entire dynamic range of the detector and to avoid saturation of the fluorescent signal. Fluorescent images have been inverted using ImageJ software and representative image stacks showing Mx2tRFP expression are presented.

Video S1

Kinetics of Mx2tRFP induction in IECs grown under standard culture conditions. Time-lapse imaging of IECs Mx2tRFP stimulated with 500U/ml IFN-β (above) and 25ng/ml IFN-λ3 (below).

Video S2

Kinetics of Mx2tRFP induction in polarized IECs. IECs harboring Mx2tRFP were cultured on transwell inserts for 21 days. Time-lapse imaging of cells stimulated with 500U/ml IFN-β (above) and 20ng/ml IFN-λ3 (below).
